# Extracellular DNA: A Missing Link in the Pathogenesis of Ectopic Mineralization

**DOI:** 10.1002/advs.202103693

**Published:** 2021-12-23

**Authors:** Min‐juan Shen, Kai Jiao, Chen‐yu Wang, Hermann Ehrlich, Mei‐chen Wan, Dong‐xiao Hao, Jing Li, Qian‐qian Wan, Lige Tonggu, Jian‐fei Yan, Kai‐yan Wang, Yu‐xuan Ma, Ji‐hua Chen, Franklin R. Tay, Li‐na Niu

**Affiliations:** ^1^ National Clinical Research Center for Oral Diseases & State Key Laboratory of Military Stomatology & Shaanxi Key Laboratory of Stomatology, School of Stomatology The Fourth Military Medical University Xi'an Shaanxi 710032 P. R. China; ^2^ Institute of Electronic and Sensor Materials TU Bergakademie Freiberg Freiberg 09599 Germany; ^3^ Center for Advanced Technology Adam Mickiewicz University Poznan 61‐614 Poland; ^4^ Department of Applied Physics Xi'an Jiaotong University Xi'an Shaanxi 710049 P. R. China; ^5^ School of Medicine University of Washington Seattle WA 98195 USA; ^6^ The Dental College of Georgia Augusta University Augusta GA 30912 USA

**Keywords:** amorphous calcium phosphate, biomineralization, collagen, ectopic calcification, extracellular nucleic acids

## Abstract

Although deoxyribonucleic acid (DNA) is the genetic coding for the very essence of life, these macromolecules or components thereof are not necessarily lost after a cell dies. There appears to be a link between extracellular DNA and biomineralization. Here the authors demonstrate that extracellular DNA functions as an initiator of collagen intrafibrillar mineralization. This is confirmed with in vitro and in vivo biological mineralization models. Because of their polyanionic property, extracellular DNA molecules are capable of stabilizing supersaturated calcium phosphate solution and mineralizing 2D and 3D collagen matrices completely as early as 24 h. The effectiveness of extracellular DNA in biomineralization of collagen is attributed to the relatively stable formation of amorphous liquid droplets triggered by attraction of DNA to the collagen fibrils via hydrogen bonding. These findings suggest that extracellular DNA is biomimetically significant for fabricating inorganic–organic hybrid materials for tissue engineering. DNA‐induced collagen intrafibrillar mineralization provides a clue to the pathogenesis of ectopic mineralization in different body tissues. The use of DNase for targeting extracellular DNA at destined tissue sites provides a potential solution for treatment of diseases associated with ectopic mineralization.

## Graphical abstract

The unanticipated function of extracellular DNA as mineralization initiators is presented from biological and materials science perspectives. Rapid matrix calcification is accomplished by infiltration of DNA‐stabilized liquid precursors into collagen fibrils. This serendipitous discovery enables facile preparation of biomimetic collagen‐based materials for tissue regeneration and targeting extracellular DNA for treatment of diseases associated with ectopic calcification.

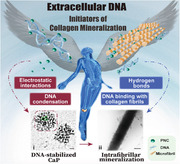

## 1 Introduction

Research on the properties of nucleic acids has never ceased since the discovery of the double helix structure of deoxyribonucleic acids (DNA) in 1868.^[^ ^1^ ^]^ Over the past 150 years, investigations in the functions of DNA have proceeded from the cellular level to the molecular level. It is now known that DNA molecules are not merely carriers of genetic information and are not only confined to the cell nucleus. The extracellular forms of these biopolymers have extensive applications in nanotechnology and bioengineering.^[^ ^2^ ^]^ Among these applications, the link between DNA and biomineralization is particularly intriguing and has not been explored in depth. Early evidence showed that DNA were reasonably well‐preserved in archaic skeletal remains.^[^ ^3^ ^]^ Recent evidences identified extracellular DNA in microscopical calcifications derived from atherosclerotic plaques,^[^ ^4^, ^5^ ^]^ gallstones,^[^ ^6^ ^]^ and breast cancer,^[^ ^7^ ^]^ suggesting that free forms of these biopolymers are likely to be involved in ectopic calcium phosphate mineralization. All these cues point to the potential of extracellular DNA as a biomineralization initiator for inducing highly ordered calcium phosphate deposition within an organic matrix.

Biomineralization is a ubiquitous and tightly regulated process in vertebrates. This process utilizes biomacromolecules to initiate nucleation of inorganic minerals, creating hierarchical skeletal components for supportive function and calcium storage.^[^ ^8^ ^]^ Intrafibrillar mineralization of collagen accounts for the unique structural and mechanical properties of bone. In the last decade, scientists have been actively searching for biomimetic molecules as substitutes for noncollagenous proteins which can mineralize collagen intrafibrillarly by preventing premature crystallization from supersaturated calcium and phosphate (CaP)‐containing solution. This enables infiltration of amorphous calcium phosphate (ACP) into collagen fibrils to produce nanostructured collagen‐apatite composites.^[^ ^9^ ^]^ Having the chemical properties of polyanions, DNA possesses the capacity to bind nonspecifically to calcium ions.^[^ ^9^, ^10^ ^]^ Although DNA has frequently been identified outside cells,^[^ ^11^, ^12^, ^13^, ^14^ ^]^ extracellular DNA has never been used for intrafibrillar collagen mineralization at the molecular level.^[^ ^9^, ^15^ ^]^

Here, we hypothesize that extracellular DNAs participate in intrafibrillar collagen mineralization. To test this hypothesis, we utilize in vitro and in vivo models to demonstrate that extracellular DNA could stabilize supersaturated CaP solution and induce intrafibrillar mineralization of 2D and 3D collagen matrices within 24 h. To establish the effectiveness of DNA‐ACP in directing collagen intrafibrillar mineralization, we employed chemo‐analytical techniques and cryogenic electron microscopy (cryo‐EM) to characterize the stability of DNA‐ACP and examine the morphology of collagen fibrils mineralized by DNA‐ACP. We resorted to molecular dynamics (MD) simulation to provide detailed modeling of the mineralization process at the molecular scale. Finally, we adopted an in vivo intramuscular implantation animal model to authenticate that extracellular DNA serves as a novel therapeutic target for the management of ectopic mineralization. The present research establishes a new role of extracellular DNA as mineralization initiators and offers a potential solution for targeting extracellular DNA for the management of ectopic calcification diseases.

## 2 Results and Discussion

**Figure 1:**
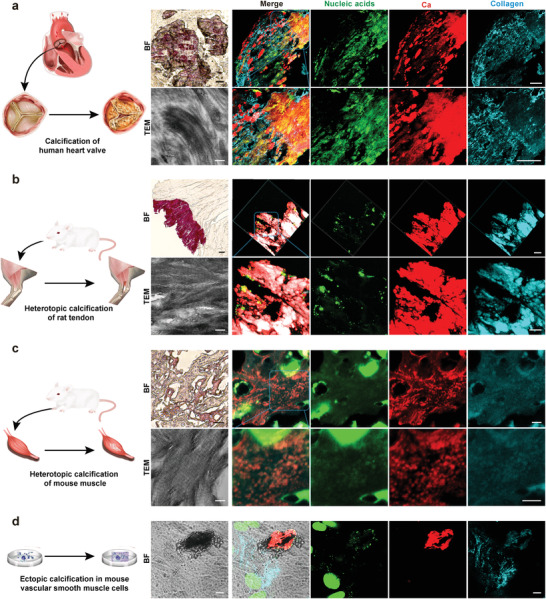
Extracellular DNA deposition identified from pathological collagen mineralization models in vivo and in vitro. a) Bright field (bar: 50 µm) and CLSM images (bar: 100 µm) taken from a human heart valve that was calcified in vivo showing the presence of randomly scattered extracellular DNA within the mineralized tissue. TEM image taken from the same specimen showing intrafibrillar mineralization of collagen fibrils within the tissues (bar: 200 nm). b) Bright field (bar: 50 µm) and CLSM images (bar: 20 µm) taken from an ectopically calcified rat Achilles tendon in vivo showing the presence of extracellular DNA within the mineralized tissue. TEM image taken from the same specimen showing intrafibrillar mineralization of collagen fibrils within the tissues (bar: 200 nm). c) Bright field (bar: 50 µm) and CLSM images (bar: 5 µm) taken from a bone‐like intramuscular ectopic calcification in vivo showing the presence of extracellular DNA within the mineralized tissue. TEM image taken from the same specimen showing intrafibrillar mineralization of collagen fibrils within the tissue (bar: 200 nm). d) Bright field (bar: 10 µm) and CLSM images (bar: 10 µm) taken from a mineralized nodule secreted by mouse vascular smooth muscle cells that were cultured in osteogenic medium in vitro. Similar to the three in vivo models, extracellular DNA was identified within the ectopically mineralized nodule.

We first employed three pathological calcification models (the human heart valve calcification model, the murine intramuscular ectopic calcification model, and the rat Achilles tendon calcification model) to investigate the correlation between nucleic acid deposition and ectopic mineralization of the extracellular matrix (ECM). Histological sections of prospective ectopic calcification were examined with immunofluorescence staining and confocal laser scanning microscopy (CLSM) by superimposition of nucleic acids (mainly DNA), mineral phases, and collagen fibrils in the regions of calcification. Extracellular DNA deposition was apparent within the ECM of heart valve calcifications (**Figure** **1**a). Likewise, CLSM of the sections prepared from the rat Achilles tendon calcification model demonstrated regions of calcified collagen that coincided with extracellular DNA deposition (Figure 1b). Examination of ectopic bone‐like intramuscular calcifications also identified extracellular DNA within the mineralized tissue (Figure 1c). Transmission electron microscopy (TEM) of specimens retrieved from the aforementioned calcification models showed that collagen fibrils within the calcified regions were heavily mineralized via intrafibrillar mineralization. Intrigued by these in vivo observations, we further utilized mouse vascular smooth muscle cells that had been cultured in osteogenic medium for identification of ectopically mineralized nodules in vitro.^[^ ^12^ ^]^ Extracellular DNA was also detected by immunofluorescence staining in the calcified sites (Figure 1d and Movie S1, Supporting Information).

**Figure 2:**
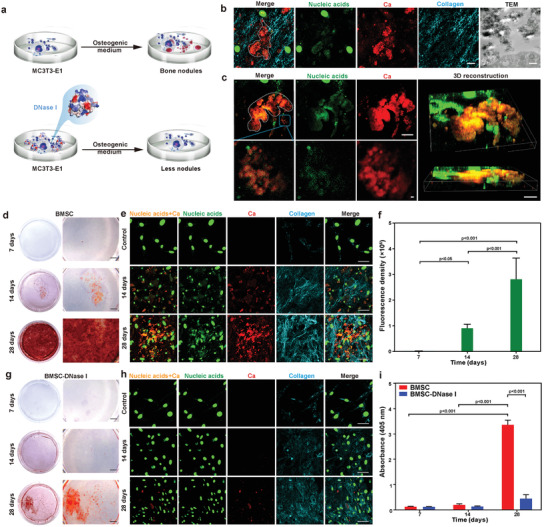
Correlation of extracellular DNA deposition with ECM calcification of osteoblast‐like MC3T3‐E1 cells cultured in osteogenic medium in vitro. a) Scheme of MC3T3‐E1 cells cultured in osteogenic medium with or without incorporation of DNase I. b) CLSM images of bone‐like nodules formation (white dashed line) in MC3T3‐E1 cells for 21 days. Extracellular DNA deposited at sites of bone‐like nodules (bar: 10 µm). TEM image taken from the same specimen showed calcified extracellular matrix (bar: 500 nm). c) Confocal live imaging of mineralized nodules (white dashed line) in MC3T3‐E1 cells（bar: 10 µm）. Colocalization of extracellular DNA and bone‐like nodules are clearly identified in the high magnification images of the blue rectangle in (c) (bar: 2 µm). 3D reconstruction showed extracellular DNA aggregation within the regions of calcification (bar: 20 µm). d,e) Alizarin red S staining (d, bar: 2 mm) and CLSM analysis (e, bar: 50 µm) of mineralized MC3T3‐E1 cells cultured in osteogenic medium for 7, 14, and 28 days. f) Immunofluorescence of extracellular nucleic acids was performed on mineralized MC3T3‐E1 cells stained with SYTOX Green. The data were analyzed quantitatively. Means ± standard deviations (*n* = 6), one‐way ANOVA. There was increase in extracellular DNA deposition over time. g,h) Alizarin red S staining (g, bar: 2 mm) and CLSM analysis (h, bar: 50 µm) of MC3T3‐E1 cells cultured in osteogenic medium containing DNase I. Extracellular DNA was barely observed. i) Semiquantitative analysis of alizarin red S stained particles harvested from the MC3T3‐E1 group and the MC3T3‐E1‐DNase I group. Extracted solution was measured at 405 nm. Means ± standard deviations (*n* = 3), two‐way ANOVA.

Identification of the association between extracellular DNA and ectopic calcification in both the in vivo and in vitro pathological calcification models spurred us to explore whether such a relationship also exists in osteoblast‐mediated matrix mineralization. This was achieved by evaluation of osteoblast‐like MC3T3‐E1 cells that had been cultured in osteogenic medium.^[^ ^16^ ^]^ Abundant extracellular mineralized nodules were identified after these cells were cultured for 21 days. Collagen, minerals and extracellular DNA were observed within the mineralized nodules using immunofluorescence staining and CLSM; the location of extracellular DNA overlapped with the areas of calcification (**Figure** **2**b). TEM identified that collagen fibrils within the calcified regions were mineralized heavily via intrafibrillar mineralization (Figure 2b). Confocal time‐lapse live cell imaging and 3D reconstruction provided high‐resolution images of the existence of extracellular DNA within the mineralized nodules secreted by live MC3T3‐E1 cells that had been cultured in osteogenic medium for 21 days (Figure 2c).

To gain an in‐depth understanding of the relation between extracellular DNA and ECM calcification, extracellular mineralization by MC3T3‐E1 cells cultured in osteogenic medium for 7, 14, and 28 days were compared using alizarin red S staining for mineralization and CLSM for DNA detection. The extent of calcification was significantly increased at 28 days, compared with cells that were cultured for 14 and 7 days (*p* < 0.001). The extracellular DNA content also increased at 28 days compared with what was identified at 14 and 7 days (*p* < 0.001) (Figure 2d–f,i). When MC3T3‐E1 cells were treated with deoxyribonuclease (DNase I), degradation of the extracellular DNA resulted in significant reduction of ECM calcification at 28 days (*p* < 0.001; Figure 2g–i). These finding suggests that extracellular DNA has the potential to accelerate matrix calcification.

Cell death that occurs in pathological calcification such as urolithiasis,^[^ ^17^ ^]^ osteoarthritis,^[^ ^18^ ^]^ cholelithiasis,^[^ ^19^ ^]^ nephrocalcinosis,^[^ ^20^ ^]^ and atherosclerosis^[^ ^21^ ^]^ contributes to the release of extracellular nucleic acids. The cation‐chelating capability of extracellular DNA molecules enable them to bond to proteins with electrically charged amino acid moieties in the ECM.^[^ ^22^ ^]^ This, in turn, results in the formation of stable aggregates that prevent DNA degradation.^[^ ^4^ ^]^ The existence of extracellular DNAs in body fluids may be accountable for pathological calcification. Based on our observation of the association between extracellular DNA with ECM calcification, it is reasonable to consider whether deposition of extracellular DNA on ECM collagen will induce intrafibrillar collagen mineralization and initiate matrix calcification.

**Figure 3:**
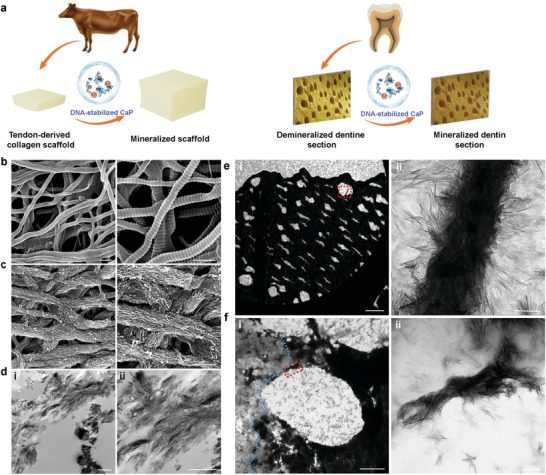
Mineralization of 3D collagen fibril models by DNA‐stabilized CaP. a) Scheme of the preparation and mineralization of 3D collagen fibril models. SEM images of b) nonmineralized and c) mineralized bovine Achilles tendon‐derived pristine collagen scaffolds (bars: 500 nm). d) TEM images showing the intrafibrillar mineralization of the collagen scaffolds induced by DNA‐stabilized CaP. d[i]) Low magnification image (bar: 2 µm). d[ii]) High magnification image of heavily mineralized collagen fibrils. Some of the mineralized fibrils demonstrated cross‐banding (bar: 500 nm). e) TEM images of thin cryogenic sections (200 nm thick) of demineralized human dentin that had been remineralized with DNA‐stabilized CaP. e[i]) Low magnification image (bar: 10 µm). e[ii]) High magnification image of the area indicated by the red rectangle showing extensive intra/extrafibrillar mineralization of the dentin collagen fibrils (bar: 100 nm). f) TEM images of cryogenic thin sections of demineralized dentin in the process of remineralization. f[i]) Partially remineralized dentinal tubules (bar: 2 µm). Increased electron density on the right of the blue dotted line is indicative of the presence of remineralized collagen fibrils. f[ii]) High magnification image of the intra/extrafibrillar remineralization indicated by the red‐dotted rectangle in (f–i) (bar: 200 nm).

To demystify this conundrum, reconstituted collagen scaffolds prepared from bovine Achilles tendon and 200 nm thick cryogenic sections of demineralized human dentin were used as in vitro 3D collagen matrix models. Total DNA isolated from MC3T3‐E1 cells was used to stabilize CaP in a supersaturated mineralization medium comprising 3.5 mm CaCl_2_·2H_2_O and 2.1 mm K_2_HPO_4_ to produce DNA‐stabilized CaP. Stabilization without precipitation occurred when the DNA concentrations were higher than 180 µg mL^−1^ (Figures S3 and S4, Supporting Information). DNA‐stabilized CaP at pH 7.0 was used as the mineralization medium, which was incubated with the 3D collagen scaffolds for 5 days. Scanning electron microscopy (SEM) and TEM examination of the bovine Achilles tendon‐derived collagen scaffolds and demineralized human dentin showed that those matrices were heavily mineralized/remineralized, with evidence of both intrafibrillar and extrafibrillar mineralization (**Figure** **3** and Figure S5, Supporting Information).

**Figure 4:**
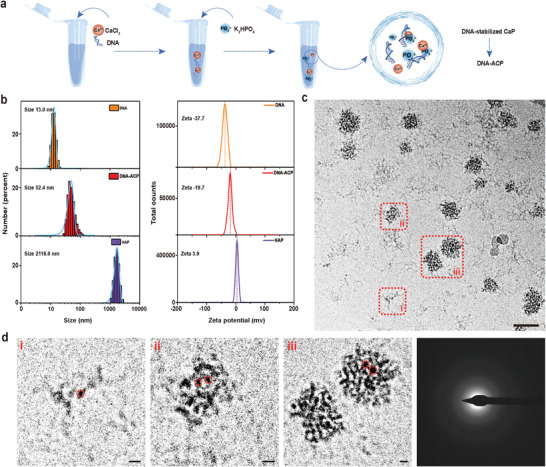
Characterization of DNA‐ACP. a) Scheme of DNA‐ACP preparation. b) Hydrodynamic diameter distribution (left) and zeta potential (right) of nucleic acids extracted from cells and DNA‐ACP. From top to bottom: DNA, DNA‐ACP, and hydroxyapatite (HAP). c) Cryogenic electron microscopy of DNA‐ACP taken at 60 min after preparation (bar: 50 nm). d) High magnification image of the areas indicated with red‐dotted rectangles in (c), showing the different growth stages of DNA‐ACP (bars: 5 nm). Electron‐dense nanometer‐sized prenucleation clusters (highlighted by red circles) that aggregated to form DNA‐ACP. The amorphous nature of the DNA‐ACP was validated by selected area electron diffraction.

Because DNA‐stabilized CaP was involved in directing collagen mineralization, different chemoanalytical methods were used to characterize those entities. As measured by dynamic light scattering, the mean hydrodynamic diameter of DNA‐stabilized CaP was 52.4 nm (**Figure** **4**b). Conversely, the size of CaP without DNA addition was 2116 nm; the CaP precipitated as a crystalline phase. The zeta potential of pristine DNA was −37.7 mV. After adding calcium and phosphate ions, the zeta potential changed to −19.7 mV for DNA‐stabilized CaP (Figure 4b). High‐angle annular dark‐field scanning TEM combined with elemental mapping and selected area electron diffraction (SAED) confirmed the presence of amorphous DNA‐stabilized CaP (DNA‐ACP) phase. The distribution of DNA‐derived nitrogen overlapped with the distributions of elemental calcium and phosphorus within those particulates (Figure S6, Supporting Information).

cryo‐EM was used to examine the morphology of DNA‐ACP in their hydrated status. The DNA‐ACP existed in solution as electron‐dense nanoparticulates (Figure 4c). These amorphous nanoparticles consisted of prenucleation clusters with sizes <5 nm in diameter (Figure 4c,d).^[^ ^23^, ^24^ ^]^ They remained stable after 8 h without transforming into crystalline CaP phases (Figure S7, Supporting Information). These results confirmed that polyanionic nucleic acids were capable of stabilizing saturated CaP to form prenucleation nanoclusters and ACP.

**Figure 5:**
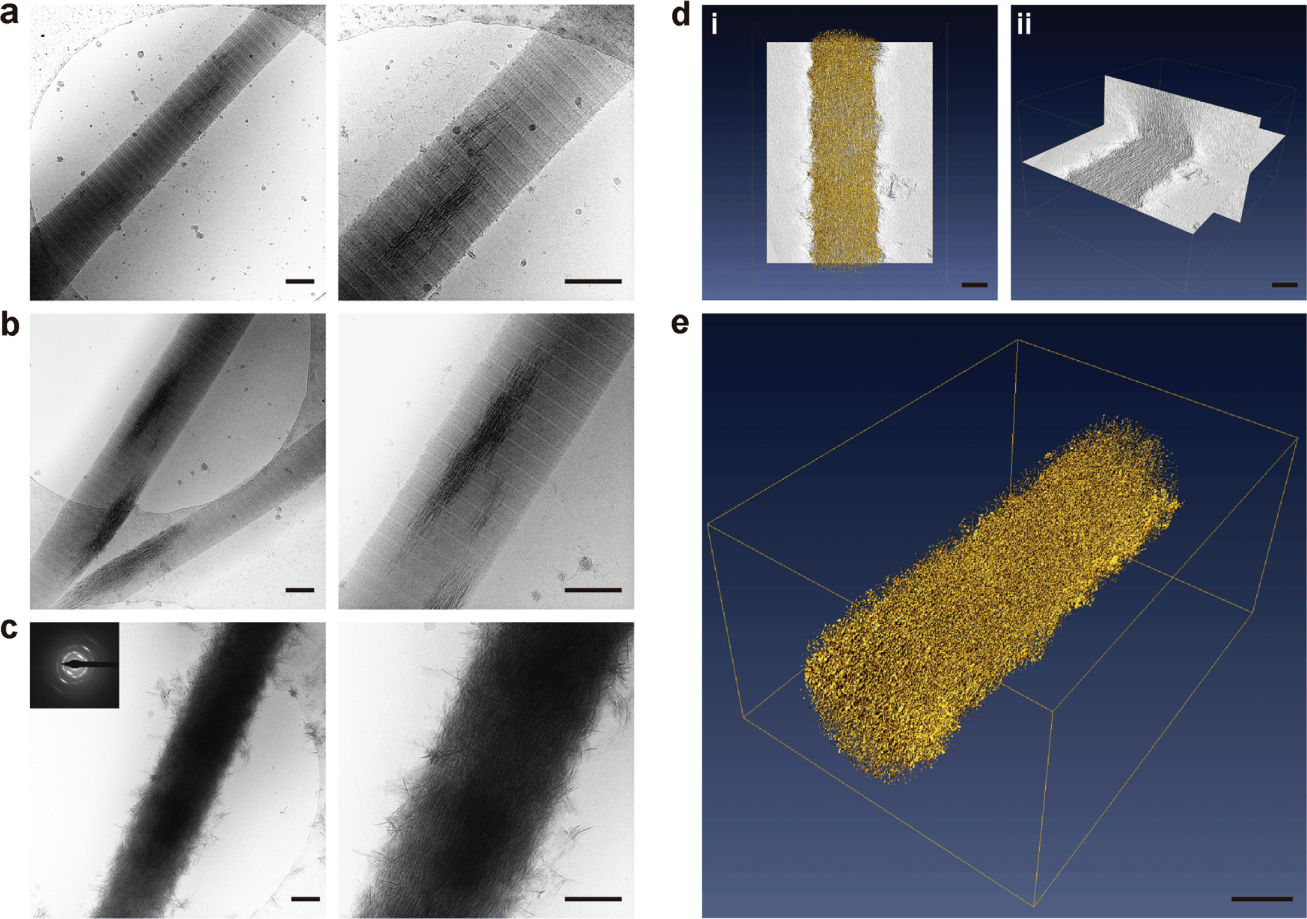
Cryogenic electron microscopy images of different stages of collagen mineralization and cryogenic electron tomography of collagen fibrils mineralized by DNA‐ACP. a–c) Unstained, reconstituted collagen fibrils that had been immersed in DNA‐ACP medium for 3, 5, and 24 h (bars: 200 nm). Minerals began to form within the fibrils after 3 h. At 24 h, complete intrafibrillar and extrafibrillar mineralization were achieved with apatite crystallites (indicated by SAED insert). d,e) 3D reconstruction from an electron tomography tilt series of DNA‐directed intrafibrillar mineralization (bars: 200 nm; movie available as Movie S2, Supporting Information). d[i]) Surface rendering of a collagen fibril that was mineralized for 24 h. d[ii]) Segmentation of the 3D volume to illustrate intrafibrillar and extrafibrillar mineralization (movie available as Movie S3, Supporting Information). e) 3D rendering of a heavily mineralized collagen fibril at 24 h.

Although DNA‐ACP was capable of mineralizing 3D collagen matrices within 5 days, the effectiveness of DNA‐ACP in directing collagen intrafibrillar mineralization has not been elucidated. Consequently, reconstituted rat tail tendon‐derived type I collagen fibrils were used to create an in vitro single‐layer 2D mineralization model. The collagen fibrils were examined with cryo‐EM to monitor the evolution of mineralization mediated by DNA‐ACP in their native hydrated state. Intrafibrillar minerals were seen along the C‐axis of the collagen fibrils as early as 3 h after the assembled fibrils were immersed into a freshly prepared mineralization medium (**Figure** **5**a). More extensive intrafibrillar mineralization was identified after 5 h (Figure 5b and Figure S8, Supporting Information) and mineralization of the entire fibril was accomplished as early as 24 h (Figure 5c and Figure S9 and Movie S2, Supporting Information). 3D reconstruction of a tilt series obtained by cryogenic electron tomography of a completely mineralized fibril at 24 h revealed the presence of both intrafibrillar and extrafibrillar minerals within the fibril (Figure 5d,e and Figure S10 and Movie S3, Supporting Information).

This is the first time complete in vitro mineralization of pristine collagen fibrils is reported within such a short time. Although complete intrafibrillar mineralization of collagen fibrils within 24 h had been reported previously, those in vitro models utilized collagen fibrils that contained additional positive/negative charges.^[^ ^25^, ^26^ ^]^ Our in vitro findings provided compelling evidence that nucleic acids deliver a strong driving force in facilitating ACP infiltration into collagen fibrils, which results in rapid mineralization.

**Figure 6:**
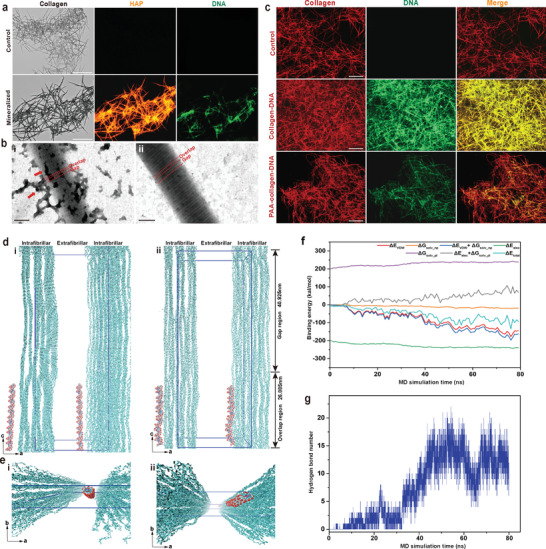
Characterization and molecular dynamics (MD) simulations of the binding between DNA and collagen fibril. a) Bright field and CLSM images of collagen fibrils mineralized by DNA‐ACP after 5 days (bar: 75 µm). Apatite was labeled with alizarin red S and emitted orange–red fluorescence. DNA was labeled with SYTOX Green and emitted green fluorescence. A moderate amount of DNA adsorbed on the mineralized collagen fibrils. b) TEM of ruthenium red and uranyl acetate‐stained collagen fibrils (bar: 200 nm). DNA was labeled by ruthenium red. b[i]) Electron‐dense aggregates representing DNA attracted to the gap and overlap zones of a collagen fibril after 48 h of incubation (red arrows). b[ii]) A pristine collagen fibril without incubation with DNA. c) CLSM images of DNA binding with collagen fibrils (bar: 50 µm). DNA (green) bound to pristine collagen fibrils (middle, red) and to anionic polyacrylic acid‐modified collagen fibrils (bottom, red). d) A snapshot showed 64 base pair‐double stranded DNA (64 bp‐dsDNA) was placed in the extrafibrillar regions (mainly located at the overlap region) and 2.5 nm away from the collagen microfibrillar structures (left, side view). The blue lines indicate the 67‐nm‐long simulation box with the dimensions of *a* = 24.2 nm, *b* = 5.66 nm, and *c* = 67.79 nm with *α* = 90°, *β* = 90°, and *γ* = 105.58°. MD simulation (*t* = 80 ns) showing binding of dsDNA to collagen fibrils (right, side view). Cyan ribbons represent collagen triple helices and red ribbons represent 64 bp‐dsDNA molecules. e) Perspective view snapshots of 64 bp‐dsDNA binding with collagen microfibrillar structure. f) Binding energy between 64 bp‐dsDNA and collagen (*t* = 80 ns). van der Waals energy (Δ*E*_vdw_) and nonpolar solvation energy (Δ*G*_solv_np_) are favorable for DNA interaction with collagen fibrils. g) Formation of multiple hydrogen bonds between 64 bp‐dsDNA and collagen (*t* = 80 ns).

With the use of CLSM, we observed that nucleic acids were adsorbed on the collagen fibrils even after they were completely mineralized (**Figure** **6**a). This finding suggested that DNA‐collagen interaction might exist during CaP mineralization. Accordingly, reconstituted collagen fibrils were incubated with DNA for 48 h and stained with ruthenium red and uranyl acetate for TEM. Ruthenium red‐stained, electron‐dense filamentous aggregates were attached randomly along the surface of the collagen fibril (Figure 6b). Based on the concept of electrostatic attraction, the negatively charged DNA should be attracted to and localized around the positively charged regions on the collagen fibrils, such as the a and c bands.^[^ ^27^ ^]^ Thus, the driving forces for binding of DNA to collagen likely involve interactions other than Coulombic attraction.^[^ ^28^ ^]^ To further investigate the interactions involved in binding of DNA with collagen, we resorted to the use of anionic collagen models for analysis. Images taken with CLSM indicated that the fluorescence intensity of DNA decreased only slightly when these molecules interacted with anionic polyacrylic acid‐bound collagen. This suggests that the ion association is not the only driving force in DNA‐collagen interaction (Figure 6c).

Fourier transform‐infrared spectroscopy was subsequently used to probe the DNA‐collagen interaction at the molecular level (Figure S11a, Supporting Information). The broad bands displayed at 3000–3700 cm^−1^ in the infrared spectrum of freeze‐dried DNA‐treated collagen were assigned to O—H and N—H stretching bands. This is indicative of the strength of the hydrogen bonds (Figure S11a,b, Supporting Information).^[^ ^29^ ^]^ Collagen fibrils contain polar side‐chains such as —NH_2_ and —COOH groups.^[^ ^26^ ^]^ The nucleotides in DNA are capable of participating in hydrogen bonding and each nucleotide can be a hydrogen‐bond donor or a hydrogen‐bond acceptor.^[^ ^30^ ^]^ To prove that hydrogen bonds are formed between collagen and nucleic acids, we employed MD simulation to provide detailed microscopic modeling at the molecular scale. The MD simulation confirmed that all‐atom 64 base pair‐double‐stranded DNA (64 bp‐dsDNA) binds with collagen fibrils (Figure 6d,e). Through calculation of the binding energy between DNA and collagen, we found that energy attributed to van der Waals attraction (Δ*E*_vdw_) and nonpolar solvation energy (Δ*G*_solv_np_), including hydrogen bonds are favorable for binding. Conversely, energy attributed to electrostatic interaction (Δ*E*_elec_) is counteracted by the polar solvation energy (Δ*G*_solv_pl_). Thus, it is likely that nonpolar and van der Waals interactions are the predominant contributors to the interaction between DNA and collagen fibrils at the overlap zone (Figure 6f). Hydrogen bond number analysis further established that these bonds played an important role in the binding of DNA to collagen (Figure 6g). Taken together, DNA molecules are capable of binding to collagen fibrils via hydrogen bonding, which accounts for rapid intrafibrillar collagen mineralization.

The nucleic acids used for inducing rapid collagen mineralization in the present study were total genomic DNAs extracted from cells that have high molecular weight (15‐30 kbp).^[^ ^31^ ^]^ It is not clear whether only DNA with high molecular weight can initiate collagen mineralization. Thus, different dsDNA with different molecular weight, including 41 bp (Mw: 26 kDa), 64 bp (Mw: 40 kDa), and 128 bp (Mw: 79 kDa) dsDNA, and fish sperm DNA (Mw: 523) were used for preparation of collagen mineralization medium (1 mg mL^−1^ DNA, 3.5 mm CaCl_2_·2H_2_O and 2.1 mm K_2_HPO_4_). TEM showed that 64 and 128 bp dsDNA with molecular weight higher than 40 kDa stabilized a supersaturated mineralization medium and induced collagen mineralization (Table S1 and Figure S12, Supporting Information).

It is important to note that extracellular DNAs that originate from cell necrosis are large‐sized fragments above 50 kbp.^[^ ^32^ ^]^ Cell‐free DNA derived from apoptotic DNA cleavage produces a characteristic fragmentation pattern of 160–200 bp.^[^ ^33^ ^]^ The molecular weights of these DNA entities are all beyond 40 kDa. Because the extracellular DNA fragments generated under in vivo pathological conditions possess similar characteristics as those DNA entities investigated herein, one can logically deduce that they are similarly capable of mediating extracellular calcification.

ACP has to possess the potential to flow, with little or no tendency to disperse and relatively high incompressibility for it to infiltrate into the intrafibrillar milieu of a collagen fibril. Accordingly, MD simulation with rigorous theoretical modeling approaches was used to predict, reproduce, and analyze the DNA‐ACP formation process. Addition of calcium ions to nucleic acids caused DNA aggregation and condensation (Figure S13a–c and Movie S4, Supporting Information)^[^ ^34^ ^]^ due to binding of Ca^2+^ to the polyanionic domains of the DNA. This binding resulted in reduction of electrostatic repulsion among DNA strands. At this stage, the DNA‐Ca^2+^ underwent liquid–liquid phase separation in solution. Subsequent addition of phosphate ions produced prenucleation clusters externally around the DNA aggregates. Liquid‐like DNA‐ACP was formed eventually via coalescence of the prenucleation clusters (Figure S13d–f and Movie S5, Supporting Information).

Biomineralization of nacre or bone is commonly guided by highly charged biopolymers.^[^ ^35^ ^]^ These biopolymers trigger liquid–liquid demixing of calcium carbonate or CaP prenucleation clusters into amorphous liquid droplets. These liquid droplets have been coined polymer‐induced liquid precursors.^[^ ^36^ ^]^ dsDNA or nucleic acid‐mimicking poly(adenosine diphosphate‐ribose) is capable of interacting with Ca^2+^ via their anionic domains (phosphate groups).^[^ ^37^, ^38^ ^]^ Intracellular nucleic acids have been reported to undergo liquid–liquid phase separation when triggered by electrostatic interaction.^[^ ^39^, ^40^ ^]^ Such a process is indispensable for maintenance of physiological cellular activities. The present results indicate that Ca^2+^ induces DNA condensation via electrostatic interaction at the very first few seconds.^[^ ^34^ ^]^ This is the first time we utilize an in vivo biological phenomenon (DNA condensation) to stabilize CaP mineralization precursors via liquid–liquid phase separation. Based on these findings, we surmised that introduction of DNA‐ACP to soft connective tissues such as skin and muscle induces ectopic calcification in vivo.

**Figure 7:**
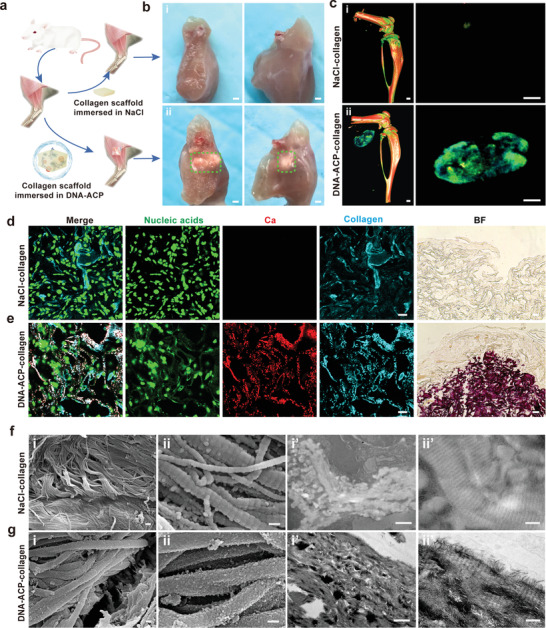
DNA‐ACP induced intramuscular ectopic calcification in vivo. a) Scheme of the surgical operation in the in vivo murine intramuscular ectopic calcification experiment. b) Photograph of intramuscular ectopic calcification samples at 3 weeks post implantation (bars: 1 mm). c) 3D reconstruction of micro‐CT scans of the ectopic calcification samples (bars: 0.5 mm). b[i],c[i]) There was no sign of collagen mineralization in the NaCl control. b[ii],c[ii]) The DNA‐ACP group showed evident ectopic calcification in the muscle tissue. d) CLSM images of the NaCl control group showed no calcium deposition (bar: 20 µm). Bright field: optical image taken from the same specimen (bar: 50 µm). e) CLSM images of intramuscular ectopic calcification induced by DNA‐ACP collagen scaffolds (bar: 20 µm). Bright field: optical image of the same specimen (bar: 50 µm). f) SEM (f[i],[ii]) and TEM (f[i′],[ii′]) images of the sample from the NaCl control group showing unmineralized collagen fibrils (bars: i and i′: 1 µm; ii and ii′: 200 nm). g) SEM (g[i],[ii]) and TEM (g[i′],[ii′]) images of ectopic calcification induced by the DNA‐ACP‐dipped collagen scaffold. Both SEM and TEM images showed heavily extra/intrafibrillar collagen mineralization (bars: i and i′: 1 µm; ii and ii′: 200 nm).

To clarify this notion, a murine intramuscular implantation model was used to validate the aforementioned proposition. Collagen scaffolds were dipped in DNA‐ACP (1 mg mL^−1^ DNA, 3.5 mm calcium ions, 2.1 mm phosphate ions) and implanted into the intramuscular pockets of mice in vivo. Similar sized collagen scaffolds that were dipped in NaCl solution have been used as control. After 3 weeks, the implantation site showed no visible inflammatory reaction, infection or extrusion. Photographs of the dissected muscles in the DNA‐ACP group unambiguously showed calcification of the collagen scaffolds. Conversely, there was no sign of collagen mineralization in the NaCl control (**Figure** **7**b). Micro‐CT and 3D reconstruction further confirmed that ectopic bone was formed in DNA‐ACP group only (Figure 7c). The CLSM images of specimens harvested from after 3 weeks of implantation showed that the DNA‐ACP collagen scaffolds were intensely calcified with extracellular DNA deposition. Alizarin red S staining showed that only the DNA‐ACP collagen scaffolds were filled with minerals (Figure 7d,e). Both SEM and TEM confirmed intra/extrafibrillar mineralization of the collagen fibrils in the DNA‐ACP group (Figure 7f,g). Taken together, these data indicate that ectopic mineralization may be induced in vivo in the presence of DNA‐stabilized CaP precursors.

**Figure 8:**
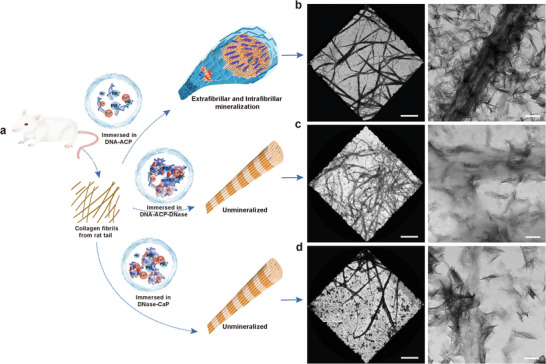
TEM images illustrating the use of DNase in inhibiting extrafibrillar and intrafibrillar mineralization of 2D collagen fibrils. a) Schematic of the experimental design. b) Grids coated with collagen fibrils were immersed into 128 bp dsDNA‐ACP for 5 days. Heavy intrafibrillar and extrafibrillar mineralization of collagen fibrils were observed. c,d) Grids coated with collagen fibrils were immersed into128 bp dsDNA‐ACP‐DNase and pure DNase, respectively, for 5 days. Collagen mineralization was inhibited in both groups. Left: low resolution of collagen fibrils (bar: 10 µm). Right: high resolution of collagen fibrils (bar: 250 nm).

Although we have demonstrated that extracellular DNA is capable of inducing ectopic mineralization of body tissues in vivo, what is more important from a pathological perspective is how to prevent this from happening. Accordingly, an experiment with three groups was designed to breakthrough this critical bottleneck. In control group 1, complete collagen mineralization was achieved after 5 days when 128 bp dsDNA (1 mg mL^−1^) was used to stabilize supersaturated CaP solution into DNA‐ACP (**Figure** **8**b). In group 2, DNase I (0.73 mg mL^−1^) was added to the mineralization medium. Group 3 was designed to eliminate the effect of DNase I on collagen mineralization. Collagen mineralization was inhibited in groups 2 and 3 (Figure 8c,d). The results indicate that DNase is the powerhouse strategy in solving the issue of unwarranted ectopic collagen mineralization in the human body. Such a strategy deserves scrupulous attention in future work.

## 3 Conclusion

The results from the present work provide a platform for amalgamating two different fields of research: extracellular DNA and biomineralization. We discovered that DNA is an efficient, readily available inducer of collagen intrafibrillar mineralization, the elimination of which may be developed into a clinical strategy for preventing ectopic mineralization diseases. This may involve the use of enzymes to degrade extracellular DNA, or biomimetic cytokines and chemokines to accelerate phagocytosis of lysed cells by macrophage,^[^ ^41^ ^]^ thereby regulating the release of DNA in vivo. Identification of DNA as a stabilizer of mineralization precursors also has applications in the field of bone tissue engineering. Extracted DNA derived from animal or human cells may be used for synthesizing biomimetic inorganic–organic hybrid materials to expedite bone regeneration.

## 4 Experimental Section

### Preparation of the DNA‐Stabilized CaP Mineralization Medium

Total cellular DNA was extracted using DNeasy Blood and Tissue Kit. All samples were tested by NanoDrop2000 ultraviolet spectroscopy (Thermo Fisher Scientific) to ensure the purity and concentration of the extracted DNA. High concentrations of total DNA (180 µg mL^−1^) and dsDNA (1 mg mL^−1^) were added to CaCl_2_·2H_2_O solution (7 mm) and mixed with an equal volume of K_2_HPO_4_ solution (4.2 mm) to form DNA‐stabilized CaP (DNA‐ACP). The pH of the final mineralization medium was adjusted to 7.0. The prepared media were clear, without precipitation.

### Mineralization of Two‐Dimensional and Three‐Dimensional Fibrillar Collagen Networks

Grids coated with 2D self‐assembled collagen were floated upside down over the mineralization medium at room temperature. At the designated time‐period, the grids were washed with deionized water three times and air‐dried. Collagen scaffolds (ACE Surgical Supply) and dentin sections were immersed into mineralization medium at room temperature, with replacement of the medium every 24 h. After 5 days, the specimens were used for characterization.

### DNA‐ACP Induced Pathological Calcification In Vivo

DNA (128 bp dsDNA)‐ACP was used with a final Ca^2+^ concentration of 3.5 mm. Collagen scaffolds were dipped in 100 µL of DNA‐ACP and implanted intramuscularly in 4‐week‐old C57BL/6J mice. The mice were randomly divided into two groups (*n* = 6). In the first group, the collagen scaffolds were dipped in DNA‐ACP. In the second group, the collagen scaffolds were dipped in NaCl solution (control). The respective collagen scaffolds (5 mm in diameter) were implanted intramuscularly, in separate pockets, in the mice for 3 weeks. Radiological evaluation was conducted 3 weeks post‐implantation. During the implantation period, the mice were fed with a standard diet and had continuous access to water.

### Statistical Analysis

Statistical analysis was performed using the SPSS statistics software 21.0 (SPSS Inc., Chicago, IL, USA). All data were expressed as the mean ± standard deviation. Data acquired for each data for each assay were evaluated for their normality and homoscedasticity assumptions prior to the use of parametric statistical methods. When these assumptions were not violated, the data were analyzed with one‐factor or two‐factor analysis of variance, followed by Bonferroni's test for multiple group analysis. All quantitative experiments were repeated at least three times to ensure the validity of observations. Statistical significance was set at *α* = 0.05.

## Conflict of Interest

The authors declare no conflict of interest.

## Supporting information

Supporting Information

Supplemental Movie 1

Supplemental Movie 2

Supplemental Movie 3

Supplemental Movie 4

Supplemental Movie 5

## Data Availability

The data that support the findings of this study are available from the corresponding author upon reasonable request.
