# Extracellular DNA: A Missing Link in the Pathogenesis of Ectopic Mineralization

**DOI:** 10.1002/advs.202201368

**Published:** 2022-04-14

**Authors:** Min‐juan Shen, Kai Jiao, Chen‐yu Wang, Hermann Ehrlich, Mei‐chen Wan, Dong‐xiao Hao, Jing Li, Qian‐qian Wan, Lige Tonggu, Jian‐fei Yan, Kai‐yan Wang, Yu‐xuan Ma, Ji‐hua Chen, Franklin R. Tay, Li‐na Niu

*Adv. Sci*. **2022**, *9*, 2103693

DOI: 10.1002/advs.202103693

In the originally published version of article, there was an error in Figure 2. The correct Figure 2 can be found below. The authors apologize for any inconvenience this may have caused.

**Figure 2:**
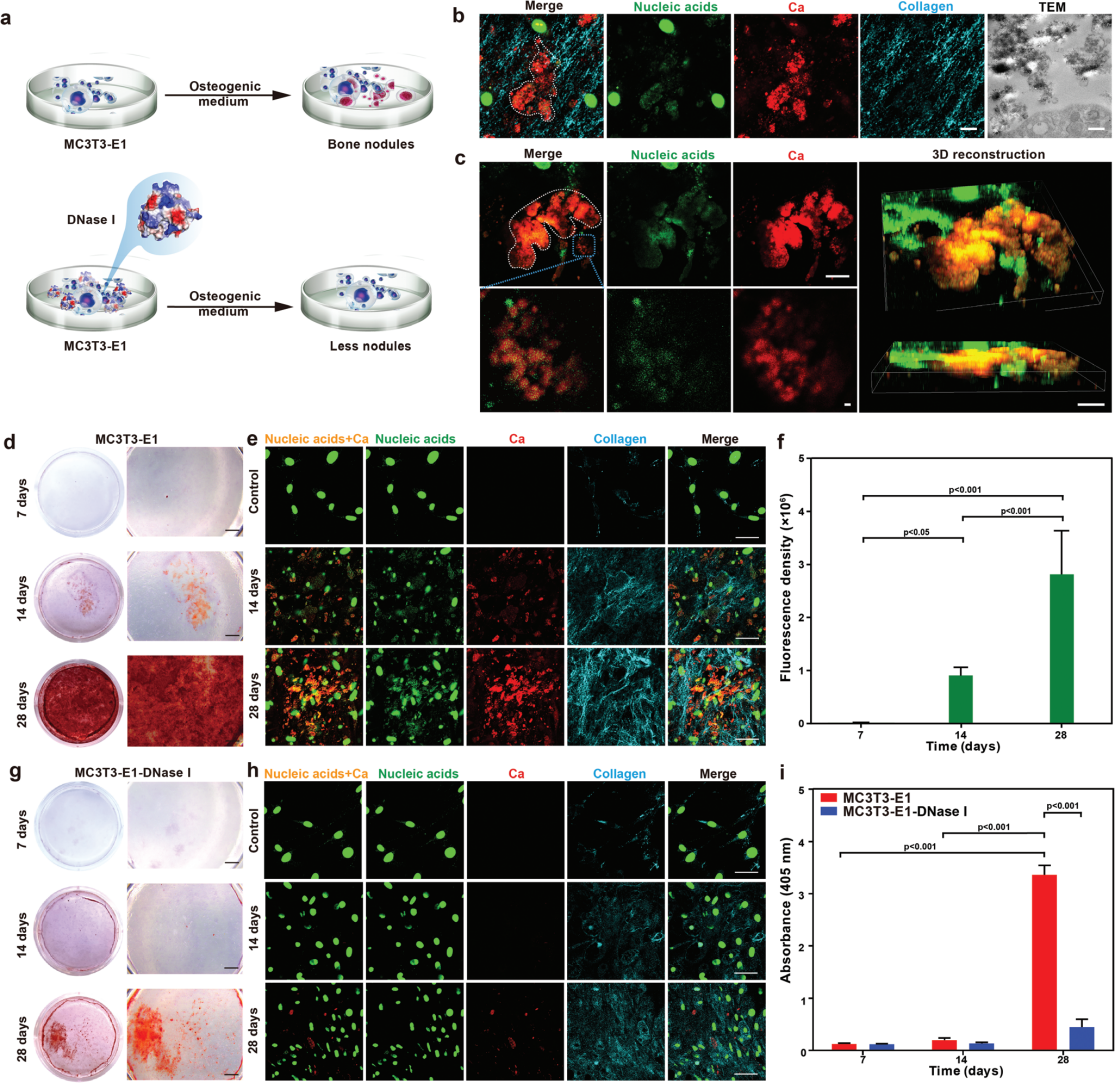
Correlation of extracellular DNA deposition with ECM calcification of osteoblast‐like MC3T3‐E1 cells cultured in osteogenic medium in vitro. a) Scheme of MC3T3‐E1 cells cultured in osteogenic medium with or without incorporation of DNase I. b) CLSM images of bone‐like nodules formation (white dashed line) in MC3T3‐E1 cells for 21 days. Extracsellular DNA deposited at sites of bone‐like nodules (bar: 10 µm). TEM image taken from the same specimen showed calcified extracellular matrix (bar: 500 nm). c) Confocal live imaging of mineralized nodules (white dashed line) in MC3T3‐E1 cells (bar: 10 µm). Colocalization of extracellular DNA and bone‐like nodules are clearly identified in the high magnification images of the blue rectangle in (c) (bar: 2 µm). 3D reconstruction showed extracellular DNA aggregation within the regions of calcification (bar: 20 µm). d,e) Alizarin red S staining (d, bar: 2 mm) and CLSM analysis (e, bar: 50 µm) of mineralized MC3T3‐E1 cells cultured in osteogenic medium for 7, 14, and 28 days. f) Immunofluorescence of extracellular nucleic acids was performed on mineralized MC3T3‐E1 cells stained with SYTOX Green. The data were analyzed quantitatively. Means ± standard deviations (*n* = 6), one‐way ANOVA. There was increase in extracellular DNA deposition over time. g,h) Alizarin red S staining (g, bar: 2 mm) and CLSM analysis (h, bar: 50 µm) of MC3T3‐E1 cells cultured in osteogenic medium containing DNase I. Extracellular DNA was barely observed. i) Semiquantitative analysis of alizarin red S stained particles harvested from the MC3T3‐E1 group and the MC3T3‐E1‐DNase I group. Extracted solution was measured at 405 nm. Means ± standard deviations (*n* = 3), two‐way ANOVA.

