# Supplementary material for: Extracellular DNA: A Missing Link in the Pathogenesis of Ectopic Mineralization
Source: Adv Sci (Weinh). 2021 Dec 23;9(5):2103693. doi: 10.1002/advs.202103693 (PMC8844461; doi:10.1002/advs.202103693)
Supplement: Supplementary file 1 — Supporting Information [file ADVS-9-2103693-s003.pdf]

© 2020 Wiley-VCH GmbH

ADVANCED SCIENCE
Open Access

Supporting Information

for *Adv. Sci.,* DOI: 10.1002/advs.202103693

Extracellular DNA: A missing link in the pathogenesis of ectopic mineralization

*Min-juan Shen, Kai Jiao, Chen-yu Wang, Hermann Ehrlich, Mei-chen Wan, Dong-xiao Hao, Jing Li, Qian-qian Wan, Lige Tonggu, Jian-fei Yan, Kai-yan Wang, Yu-xuan Ma, Ji-hua Chen, Franklin R Tay, and Li-na Niu**

Supporting Information

Extracellular DNA: A missing link in the pathogenesis of ectopic mineralization

Min-juan Shen[1,#], Kai Jiao[1,#], Chen-yu Wang[1,#], Hermann Ehrlich[2,3], Mei-chen Wan[1], Dong-xiao Hao[4], Jing Li[1], Qian-qian Wan[1], Lige Tonggu[5], Jian-fei Yan[1], Kai-yan Wang[1], Yu-xuan Ma[1], Ji-hua Chen[1], Franklin R Tay[6], Li-na Niu[1,]*

Table of Contents

SI-1. Materials and methods

SI-2. Flow chart depicting the sequence of experiments conducted in the present study [Supplementary Figure 2]

SI-3. Photograph of supersaturated CaP mineralization medium at pH 7.0 at room temperature [Supplementary Figure 3]

SI-4. SEM of supersaturated CaP mineralization medium stabilized with different concentrations of DNA [Supplementary Figure 4]

SI-5. SEM images of completely demineralized dentin thin sections that were remineralized using DNA stabilized-CaP mineralization medium for 5 days [Supplementary Figure 5]

SI-6. Characterization of DNA-CaP in the form of amorphous calcium phosphate (ACP) [Supplementary Figure 6]

SI-7. Cryogenic electron microscopy (cryo-EM) of DNA-ACP at 8 h after preparation [Supplementary Figure 7]

SI-8. Characterization of single layered collagen fibrils that had been mineralized by DNA-ACP for 5 h [Supplementary Figure 8]

SI-9. Cryo-EM images of collagen fibrils that had been mineralized by DNA-ACP for 24 h [Supplementary Figure 9]

SI-10. 3D reconstruction of a collagen fibril completely mineralized by DNA-ACP [Supplementary Figure 10]

SI-11. Binding of DNA with collagen fibrils [Supplementary Figure 11]

SI-12. TEM of collagen mineralization induced by DNA with different molecular weight [Supplementary Figure 12]

SI-13. Molecular dynamics (MD) simulation of the mechanism for DNA-ACP formation [Supplementary Figure 12]

SI-14. Captions of Supplementary Movies 1-5

SI-15. DNA sequence [Supplementary Table 1]

SI-16. Supplementary references

SI-1. Materials and methods

1. Materials

The double-stranded DNA (dsDNA) used in the present study were synthesized by Sangon Biotech (Shanghai, China). Chemicals including trichloromethane, isopropanol, ethanol, $CaCl_2 \cdot 2H_2O$, K_2HPO_4, 1-ethyl-3-(3-dimethylaminopropyl)-carbodiimide (EDC), N–hydroxysuccinimide (NHS), glycerol, acetic acid, ammonium hydroxide, ethylenediamine tetra-acetic acid (EDTA), KBr, ruthenium red, uranyl acetate, HCl, goat serum, phosphate-buffered saline (PBS), alizarin red S, glutaraldehyde, NaCl, fish sperm DNA, propylene oxide, epoxy resin, paraformaldehyde, PBS-Tween, sucrose and deoxyribonuclease I (DNase I) were all purchased from Millipore Sigma (St. Louis, MO, USA) and used as received. Dulbecco's Modified Eagle's Medium (DMEM), Alpha-MEM (α-MEM), penicillin/streptomycin, foetal bovine serum (FBS), β-glycerophosphate, ascorbic acid, dexamethasone, trizol, nuclease free water were purchased from Invitrogen (Carlsbad, USA). DNeasy Blood and Tissue Kit was purchased from Qiagen (Valencia, USA). Conventional TEM grids were acquired from Zhong Jing KeYi (Beijing, China). SYTOX™ Green nucleic acid stain was purchased from Thermo Fisher Scientific Inc. (Waltham, MA, USA).

2. Extracellular nucleic acids relevant to calcification of cells *in vitro* and tissue *in vivo*

2.1 Extracellular nucleic acid detection in MC3T3-E1 and mouse vascular smooth muscle cells (mVSMCs) of calcification in vitro

MC3T3-E1 cells and mVSMCs were purchased from American Type Culture Collection (Manassas, VA, USA). The cells were seeded on 12-well chamber slides at a density of $1x10^5$ cells/well. The MC3T3-E1 cells were cultured in α-MEM. The mVSMCs were cultured in

DMEM. After 3 days, the growth medium was replaced with osteogenic medium (growth medium supplemented with 4 mM β-glycerophosphate, 50 mg/mL ascorbic acid and 10 nM dexamethasone) to induce formation of mineralized nodules. For MC3T3 E1 cells cultured in osteogenic medium containing DNase I, the concentration of DNase I was 50 μg/mL. The osteogenic medium was replaced every 3 days. Specimens were harvested for characterization when mineralized nodules were well deposited within the chamber slides.

2.2 Live-cell imaging

The cells were washed three times with Hank's balanced salt solution and stained with SYTOX™ Green at 37 °C for 30 min. The stained cells were washed with Hank's balanced salt solution and Live Cell Imaging Solution (Thermo Fisher Scientific). For mineral staining, live cells were incubated with culture medium containing 0.01% alizarin red S solution (Wako Pure Chemical Industries, Osaka, Japan) for 15 min.[44] Live-cell imaging was performed with the confocal scanning laser microscopy (CLSM; Leica Microsystems, Wetzlar, Germany) according to the manufacturer's instruction.

2.3 Immunofluorescence

Cells seeded on 12-well chamber slides (n = 6) were fixed in 4 % paraformaldehyde and incubated with goat serum for 30 min at room temperature. The cell samples were individually exposed to collagen-I rabbit anti-mouse primary antibody (Ab34710, Proteintech, Rosemont, IL, USA) at 4 °C for 12 h. After washing, Cy3 goat anti-rabbit secondary antibodies (111-165-003, Jackson ImmunoResearch Laboratories, Inc., West Grove, PA, USA) and alizarin red S were added and incubated for 1 h in the dark. The cells were mounted in anti-fade mountant and stained with SYTOX™ Green for CLSM). For measurement of extracellular nucleic acid fluorescence intensity, areas encompassing extracellular nucleic acids in the CSLM images were manually-selected and measured via ImageJ software (National Institute of Health, Bethesda, MD, USA).[45]

2.4 Alizarin red S staining of calcification

Mineralized nodules were analyzed after the cells were cultured in osteogenic medium for a destined period (n = 3). After fixation in 4% paraformaldehyde, the cells were stained with

alizarin red S. Mineralization was quantified by colorimetric detection (NanoDrop2000 ultraviolet spectroscopy, Thermo Fisher Scientific) at 405 nm after the nodules were treated with 10% acetic acid and 10% ammonium hydroxide in 96-well plates.[46]

2.5. Animals

C57BL/6J mice and Sprague Dawley rats were purchased from the Laboratory Animal and Welfare Committee of the Air Forth Military Medical University (the Fourth Military Medical University), Xi'an, China and used for *in vivo* studies. All surgical procedures used in those experiments were approved by the ethical committee of the Fourth Military Medical University.

2.6. In vivo pathological collagen mineralization models

In the heart valve calcification model, human heart valves were obtained from volunteers with cardiac valvular calcification diseases, with informed consent and the authorization from the ethical committee of the Fourth Military Medical University. Specimens were fixed and embedded in Tissue-Tek OCT (Sakura Finetek USA, Torrence, CA, USA). Eight micrometer-thick sections were prepared using a cryogenic microtome at -20 °C.

The intramuscular ectopic calcification model was created in mice (n = 6). Mouse mesenchymal stem cells (ATCC) were seeded into 3D collagen scaffolds (ACE Surgical Supply Co., Brockton, MA, USA) under sterilized conditions.[47,48] The stem cell-containing scaffolds (5 mm x 5 mm) were implanted intramuscularly into separate pockets in 4-week-old C57BL/6J mice. The mice were euthanized after 8 weeks of calcification. Specimens were fixed and embedded in Tissue-Tek OCT. Eight micrometer-thick sections were prepared using a cryogenic microtome at -20 °C. The frozen sections were equilibrated in PBS at room temperature for 15 min to remove the "optimal cutting temperature" compound and fixed in 100% cold methanol (20 °C) for 10 min. The sections were washed in PBST (PBS + 0.01% Tween20) and blocked with PBST + 3% normal goat serum. Collagen-I rabbit anti-mouse primary antibodies (Ab34710, Proteintech) were applied to the sections overnight and incubated at 4 °C. Cy 3 goat anti-rabbit secondary antibodies (111-165-003, Jackson ImmunoResearch Laboratories), alizarin red S and SYTOX™ Green were used for fluorescence detection.

The Achilles tendon ectopic calcification model was created in rats. Four-week-old rats (n = 6) were anesthetized prior to Achilles tendon laceration surgery. The full length of each rat's

Achilles tendon was exposed. A complete transverse incision was made five times and no suturing was performed to the mid-point of the tendon. The subcuticular skin was closed by sutures.[49] After 3 months, animals were sacrificed and the Achilles tendons were collected for subsequent analysis.

3. Preparation and characterization of DNA-stabilized CaP mineralization medium

3.1 Cell culture

MC3T3-E1 murine calvarial osteoblasts (ATCC) were cultured in α-MEM that was supplemented with 10% FBS and penicillin/streptomycin in a humidified incubator with 5% CO_2 at 37 °C. When the cells reached 80% confluence, the growth medium was change to an osteogenic medium comprising 50 mg/mL ascorbic acid, 4 mM β-glycerophosphate and 10 nM dexamethasone. The osteogenic medium was changed every 3 days. For nucleic acid collection, the cells were seeded in T-75 flasks and collected after they were incubated for 14 days.

3.2 Total DNA purity and concentration

Ultraviolet spectroscopy was used to evaluate the purity and concentration of the nucleic acids. The DNA concentration was calculated using the Beer-Lambert law and the A260/A280 ratio was used to evaluate DNA purity. For DNA, an A260/280 ratio of 1.8 is generally accepted as “pure”.

3.3 Dynamic light scattering and zeta potential

The hydrodynamic diameter and the zeta potential of the DNA, DNA-CaP and CaP were measured using a Zetasizer Nano ZS detector (Malvern Instruments, Worcestershire, UK). A 4 mW He–Ne laser (633 nm) and a proper measuring angle (173°) were used for dynamic light scattering. The Smoluchowski method was used for measurement of zeta potential and the testing temperature was set at 25 °C with a voltage of 50 V. Each sample was analyzed three times.

3.4 Scanning electron microscopy (SEM) of DNA-CaP

The surface morphology of DNA-CaP was visualized using SEM (JSM-6701F, JEOL, Tokyo, Japan) operated at 15 kV. Specimens were placed on a silicon wafer, air-dried and sputter-coated with gold.

3.5 Scanning transmission electron microscopy (STEM) of DNA-ACP

The DNA-ACP was observed by STEM (JEOL 2100F) operated at 200 kV. High-angle annular dark-field scanning (HAADF-STEM) was performed. Energy dispersive X-ray spectroscopy (EDS) elemental mapping and selected area electron diffraction were also conducted to identify the mineral distribution and the crystallinity of the minerals.

3.6 Cryogenic-electron microscopy (cryo-EM) of DNA-ACP

Quantifoil Jena R2/2/gold grids coated with holey-carbon (2 μm hole size) supporting film (Electron Microscopy Sciences, Hatfield, PA, USA) were plasma-treated and used within 30 min after treatment. A 50 μL drop of DNA-ACP solution was dropped on the surface of the grids (aged for 1, 5 or 8 h after preparation). After vitrification (~1000 Å thick), the DNA-ACP coated grids were transferred to a Gatan 626 cryo-transfer holder and maintained at a temperature below -170 °C during cryo-EM observation with a Talos F200C microscope (FEI, Hillsboro, OR, USA) at 100 kV. The electron dose for each exposure was 20e $Å^{-2}$. Selected area electron diffraction was performed to confirm the crystallinity of the minerals.

3.7 Molecular dynamics (MD) simulation of DNA-ACP

To examine the polyanionic capacity of DNA, three 21 bp-dsDNA were placed in a box (a = 12.8 nm, b = 13.2 nm, c = 13.7 nm, α = 90°, β = 90°, γ = 90°), in which 252 Ca^{2+}, 384 Cl^- ions were placed. The centroid distance of DNA was 1 nm. The initial model of DNA was built by packmol and AMBER tleap, and described by GAFF2 force field and AMBER ff14SB force field. The Amber 18 molecular simulation packages were employed for performing all MD simulations with a simulation time step of 2 fs. Steepest descent and conjugate gradient algorithm energy minimization methods were introduced to remove bad contact in the initial structures using sander module of Amber 18. The entire system was minimized with a positional restraint of 10 kcal mol^{-1} $Å^{-2}$ in atoms of DNA. This was followed by equilibration at 310 K for 50 psec simulation in constant number, pressure and temperature (NVT) mode and 50 psec in constant number, pressure and temperature (NPT) mode (T = 310 K and P = 1 atm). A positional restraint of 2 kcal mol^{-1} $Å^{-2}$ in the main chain C atoms of DNA was used to relax its side chains. A 120 nsec MD simulation was performed on the systems in NPT ensemble (T = 310 K and P = 1 atm) with periodic boundary conditions. To further investigate how DNA attract HPO_4^{2-} , the

DNA, Cl^-, Ca^{2+} of the system were extracted and placed it into a box (a = 15.7 nm, b = 16.1 nm, c = 16.5 nm). This was followed by placing 252 HPO_4^{2-} ions and 504 K^+ ions into the box.

4. Mineralization of 2D and 3D fibrillar collagen networks

4.1 2D single-layer self-assembled collagen fibrils

Collagen fibrils were self-assembled from a rat tail tendon-derived collagen/acetic acid stock solution (5 mg/mL) using the dialysis method.[50] The self-assembled collagen solution was dropped on a 400 mesh Au TEM grid and dried at room temperature. A 0.3 M EDC/0.06 M NHS solution was used to crosslink and stabilized the reassembled collagen fibrils for 4 h. The grids were rinsed with deionized water for 3 times and air-dried.

4.2 3D demineralized dentin sections

Human caries-free third molars were extracted from the patients who provided informed consent for the use of the teeth for research, with approval from the ethics committee of the Fourth Military Medical University. Each tooth was immersed in 3 M HCL for 24 h to remove the minerals. Thereafter, 200 nm thick sections were obtained using a cryo-immuno diamond knife (DiATOME, CH, Switzerland) on a Leica EM UC6-NT ultracryomicrotome at −80 °C. Each section was transferred to a 200 mesh Au TEM grids. Each grid was floated on a drop of EDTA for 24 h to ensure complete demineralization. The grids were rinsed with deionized water three times and air-dried.

4.3 TEM/STEM of mineralized 2D and 3D collagen fibrils

Gold grids coated with a single layer of self-assembled collagen fibrils were immersed into DNA-stabilized CaP medium for a designated time-period. Upon retrieval, each grid was rinsed with deionized water and examined with the JEM-1230 STEM without osmication and further staining. Additional grids were examined with the STEM at 200 kV. Elemental distributions of nitrogen, oxygen, calcium and phosphate were identified via elemental mapping. Selected area electron diffraction was used to investigate the crystallinity of the deposited minerals.

Collagen scaffolds mineralized by DNA-stabilized CaP for 5 days were rinsed with deionized water, dehydrated with an ascending ethanol series (50-100 %), immersed in propylene oxide and embedded in epoxy resin. Ninety nanometer-thick sections of the collagen

scaffolds and 200 nm thick remineralized dentin sections were retrieved with copper grids and examined with the JEM-1230 TEM without osmication and further staining.

4.4 SEM of mineralized 3D collagen matrices

The surfaces of the mineralized collagen scaffolds were examined by SEM (JSM-6701F, JEOL). The specimens were dehydrated in an ascending ethanol series (50-100%), immersed in hexamethyldisilane and sputter-coated with gold prior to examination.

4.5 Cryo-EM and electron tomography of mineralized single-layer collagen

Reconstituted collagen fibrils deposited on Quantifoil Jena R2/2/gold grids with 2 μm hole size were immersed into DNA-ACP mineralization medium for designated time-periods. The grids were vitrified in the manner described in Section 3.6 without further staining. The vitrified grids were examined with a Talos F200C microscope (FEI, Hillsboro, OR, USA) at 100 kV under liquid nitrogen temperature.

For electron tomography, the selected grids were tilted in 2° steps from -50° to 50° using The University of California, San Francisco TOMO software package.[51] Alignment and 3D reconstruction of a mineralized collagen fibril were performed with the IMOD package.[52] Images were Gaussian-filtered to 10 Å. Segmentation of the 3D volume was performed with the Amira software version 2019.1 (Thermo Fisher Scientific) and Matlab version R2020a. (Mathworks, Natick, MD, USA).

5. Interaction of DNA with collagen fibrils

5.1 Attenuated total reflection-Fourier transform infrared spectroscopy (ATR-FTIR)

After the collagen fibrils were incubated with DNA for designated time-periods, the specimens were rinsed with deionized water and freeze-dried in a liquid nitrogen-cooled Emitech K775X turbo freeze dryer (Quorum Technologies Ltd, Kent, UK). The specimens were characterized using a Nicolet 6700 FTIR (Thermo Fisher Scientific) equipped with an ATR set-up. Infrared spectra were acquired over the region 4000–400 cm^{-1} with 32 scans and a resolution of 4 cm^{-1}.

5.2 *Ruthenium red staining*

Binding of DNA to collagen was examined using ruthenium red staining. TEM grids coated with collagen were floated on top of a DNA solution for 72 h. The grids were stained with 0.02 % ruthenium red, a cationic dye, to identify the collagen-bound DNA. After ruthenium red staining, the grids were counterstained with 2% uranyl acetate for 15 min prior to TEM examination.

5.3 *Immunofluorescence*

Slides coated with reconstituted type I collagen fibrils were labeled with collagen-I rabbit anti-mouse antibody (Ab34710, Proteintech) and Cy 3 goat anti-rabbit secondary antibodies (111-165-003, ImmunoResearch Laboratories, Inc.). After rinsing with PBS, the slides were immersed in DNA (1mg/mL) for 48 h. After rinsing with deionized water, the slides were labeled with SYTOX™ Green and examined by CLSM (Leica Microsystems, Wetzlar, Germany).

After the collagen fibrils were mineralized with DNA-ACP for 5 days, they were stained with alizarin red S to label the calcium ions and SYTOX™ Green for labeling the DNA. Collagen fibrils were visualized in the CLSM bright field mode to identify bound DNA on the surface of the mineralized collagen fibrils.

5.4 *Molecular dynamics simulations*

Collagen and DNA models were established to analyze the binding affinity of DNA to collagen fibrils.

5.4.1 Molecular modeling of collagen

A collagen molecular model was constructed based on the authors' previous work.[53] Briefly, full atomistic structures of collagen were constructed using homology modelling from human FASTA sequence. The FASTA sequences of α1 (I) chain (entry number NP000079) and α2 (I) chain (entry number NP000080) were obtained from PubMed. Using Modeler 9.15 (https://salilab.org/modeller/download_installation.html). The acquired FASTA sequences were aligned on the crystallography-derived collagen structures. The structure with the lowest energy was chosen based on Discrete Optimized Protein Energy calculations. The resultant triple helix

was 1.28 nm in diameter and 298.025 nm in length. Collagen fibrillar structure was described using a 67 nm long simulation box. A long collagen fibril was produced by interacting the collagen triple helix units with their self-images using periodic boundary condition, as reported in previous MD simulation studies. The distribution of mass, volume and charges within the collagen crystal structure was calculated by first dividing the triclinic crystallographic unit cell ($a = 24.2$ nm, $b = 2.83$ nm, $c = 67.79$ nm, $\alpha = 90°$, $\beta = 90°$, $\gamma = 105.58°$).

5.4.2 Molecular modeling of collagen-DNA

To investigate the interaction mechanism of collagen-DNA, 64 bp-ds DNA was placed into a unit cell ($a = 24.2$ nm, $b = 5.66$ nm, $c = 67.79$ nm, $\alpha = 90°$, $\beta = 90°$, $\gamma = 105.58°$). The DNA was placed in the extrafibrillar regions (near the overlap zone) and 2.5 nm away from the collagen fibril. In addition, 876 particles of Na^+ and 610 particles of Cl^- were included to neutralize the system. The parameters of collagen protein, DNA and water molecules were assigned with the AMBER ff14SB force field and TIP3P water model. To obtain a reasonable initial model, the system was first minimized with a positional restraint of 40 kcal mol^{-1} $Å^{-2}$ in both the DNA and collagen (5,000 steps SD method and 5000 steps CG method). Afterwards, the entire system was minimized with a positional restraint of 20 kcal $mol^{-1}Å^{-2}$ in the backbone atoms of collage and the heavy atoms of DNA (5,000 SD method steps and 5,000 steps CG method). The entire system was then energy-minimized without any restraints (5,000 steps SD method, 5,000 steps CG method). After minimization, the system was equilibrated with 2.5 nsec MD simulation NVT and 2.5 nsec NPT. Molecular dynamics simulation was performed for 80 nsec on the systems in NPT ensemble ($T = 310$ K and $P = 1$ atm) with periodic boundary conditions.

5.4.3 The molecular mechanics-generalized Born surface area (MMGBSA) method

The principle of the Molecular mechanics-Poisson–Boltzmann surface area-weighted solvent-accessible surface area (MM-PBSA-WSAS) method is well reported in the literature.[54,55] The MM-PBSA binding free energy for a ligand binding to a receptor to form a complex is expressed as:

$$\Delta G_{bind} = \Delta E_{MM} + \Delta G_{sol} - T\Delta S \quad (1)$$

where ΔE_{MM} is the change of molecular mechanics (MM) energy due to complexation in gas-phase, ΔG_{sol} is the change of solvation free energy, and $-T\Delta S$ is the change of conformational entropy upon ligand binding. E_{MM} is composed of several energy terms, including internal energies E_{int} (bond, angle, and dihedral energies), electrostatic energy E_{ele} and van der Waals interaction energy E_{VDW}. ΔG_{sol} may be further decomposed into two parts, the polar contribution (electrostatic solvation energy) which is described by the Poisson-Boltzmann continuum solvation model, and the nonpolar contribution which is described by solvent-accessible surface area (SASA). As such, the MM-PBSA binding free energy has five energy terms as shown below when the "Single Trajectory" sampling protocol[51,52] is applied:

$$\Delta G_{bind} = \Delta E_{VDW} + \Delta E_{elec} + \Delta G_{solv\_pl} + \Delta G_{solv\_np} - T\Delta S \quad (2)$$

Note that in Eq. (2), the contribution from internal energies E_{int} cancel out in the "Single Trajectory" protocol. In the present work, the polar contribution of the solvation free energy is calculated by the GB1 model using sander of Amber. The nonpolar component is calculated using a linear relationship to SASA. That is, $\Delta G_{solv\_np} = \gamma\ \Delta SASA + b$, where γ, the surface tension, is set to 0.00720 (kcal mol^{-1} $Å^{-2}$) and b to 0.0 kcal/mol, and $T\Delta S$ is the entropic contribution. The latter was neglected due to high computational costs and the lack of apparent differences with the same protein system.

6. DNA-ACP induced pathological calcification *in vivo*

6.1 Micro-computed tomography (micro-CT)

Mice at 3 weeks after implantation from each group were analyzed with a high-resolution micro-CT designed for small-animal imaging (Inveon micro-CT system Siemens AG, Munich, Germany). Each site (n = 6) was scanned at 60 kV and 148 μA. Three-dimensional reconstructions were performed using the SkyScan CtAn software (Micro Photonics, Inc., Allentown, PA, USA).

6.2 Immunofluorescence

At 3 weeks post-implantation, collagen scaffolds within the implantation sites were retrieved and sectioned at a thickness of ~10 μm using a saw microtome (SP1600, Leica,

Mannheim, Germany) for immunofluorescence analysis (n = 3). The collagen fibrils, minerals and nucleic acids were co-labeled using the method described in the Section 5.3.

6.3 TEM

Additional harvested post-implantation collagen scaffolds were then fixed with 2.5% glutaraldehyde for 3 days at 4 °C. After rinsing with PBS, the specimens were fixed in 1% osmic acid, dehydrated with an ascending ethanol series (50-100%), immersed in propylene oxide and embedded in epoxy resin for TEM characterization.

6.4 SEM

Additional harvested post-implantation collagen scaffolds were examined by SEM using the method described in the Section 4.5.

7. DNase I inhibition of collagen mineralization *in vitro*

7.1 Inhibition of collagen mineralization with DNA-ACP-DNase

TEM grids coated with self-assembled collagen were floated upside-down over the mineralization medium. The latter comprised 3.5 mM $CaCl_2.2H_2O$, 2.1 mM K_2HPO_4 and 128 bp dsDNA (1 mg/mL). DNase I (0.73 mg/mL) was added to the mineralization medium at room temperature. At the designated time-period, the grids were retrieved, rinsed and air-dried.

7.2 TEM of collagen fibrils

TEM grids coated with a single layer of self-assembled collagen fibrils were immersed into DNA-ACP-DNase medium for a designated time-period. Upon retrieval, each grid was rinsed, dried and examined with the JEM-1230 TEM without osmication and further staining.

SI-2. Flow chart depicting the sequence of experiments conducted in the present study [Supplementary Figure 2]

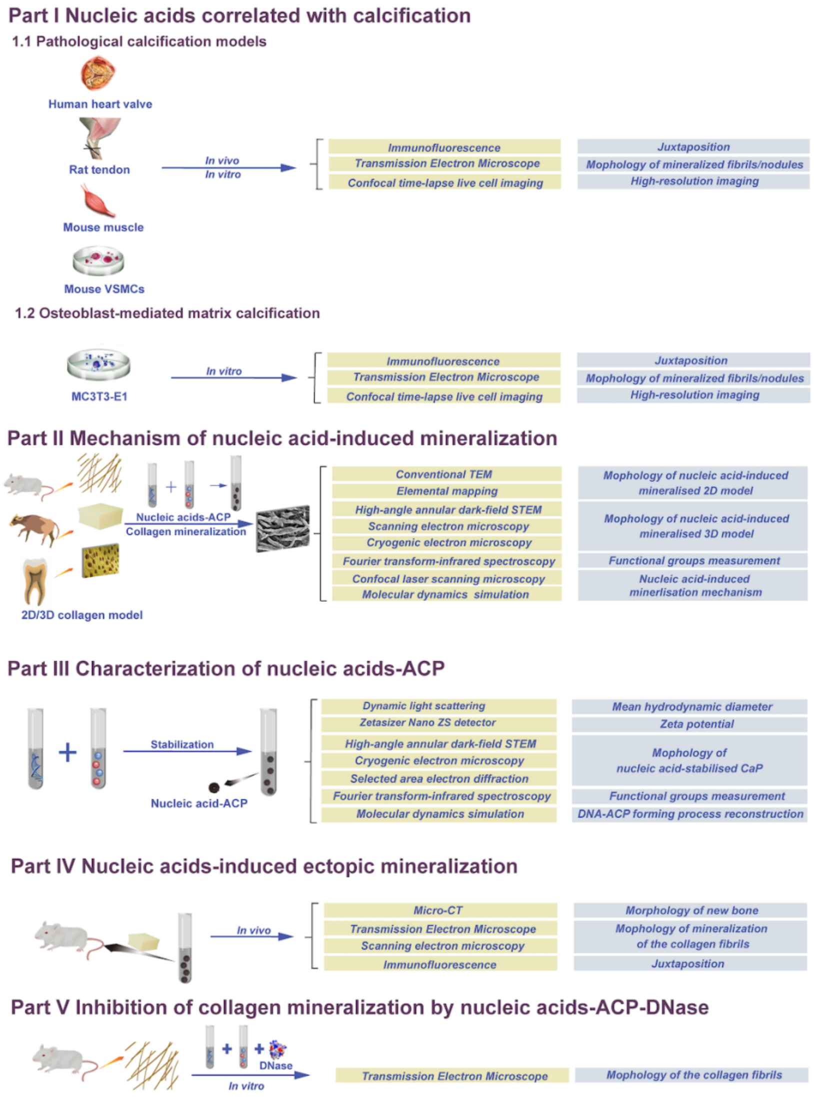

Supplementary Figure 2. Flow chart depicting the sequence of experiments conducted in the present study. Abbreviations: ACP, amorphous calcium phosphate; CaP, calcium phosphate; VSMCs, vascular smooth muscle cells; MC3T3-E1, mouse embryonic osteoblastic precursor cell; STEM, scanning transmission electron microscopy.

SI-3. Photograph of supersaturated CaP mineralization medium at pH 7.0 at room temperature [Supplementary Figure 3]

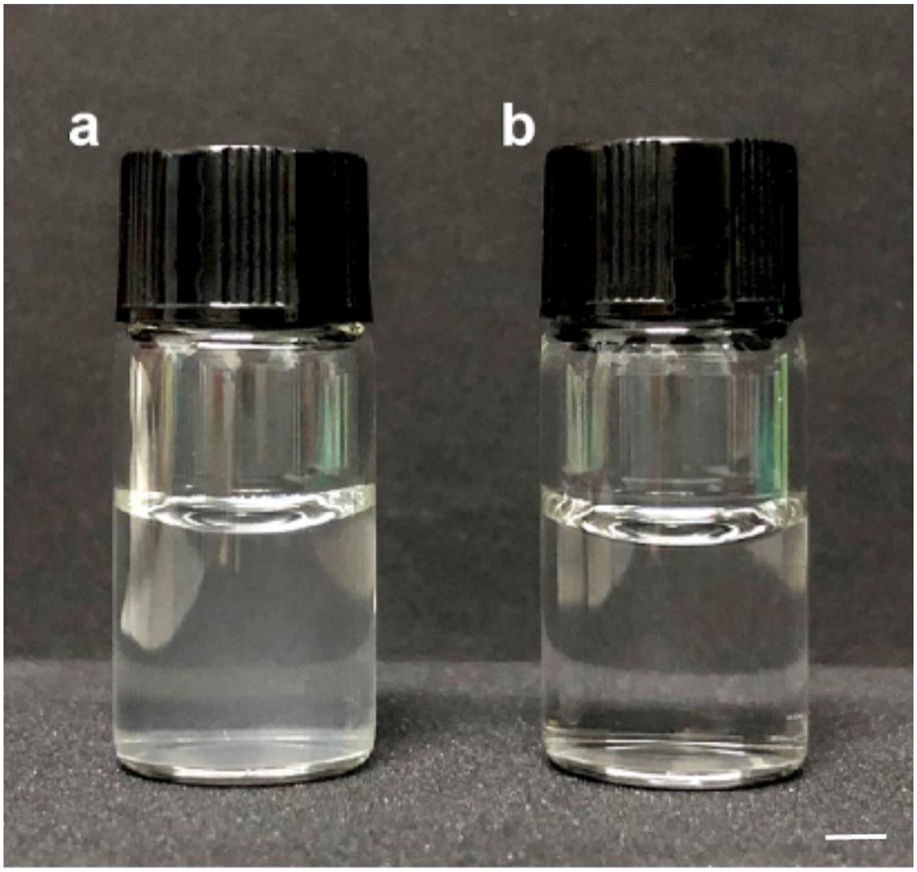

Supplementary Figure 3. Photograph of supersaturated CaP mineralization medium at pH 7.0 at room temperature. a, Turbid solution with CaP precipitation without stabilization by a nucleation inhibitor. **b,** When DNA (180 µg/mL) was used to stabilize the mineralization medium, the mixed solution remained clear for up to 120 hours (bar: 4 mm).

SI-4. SEM of supersaturated CaP mineralization medium stabilized with different concentrations of DNA [Supplementary Figure 4]

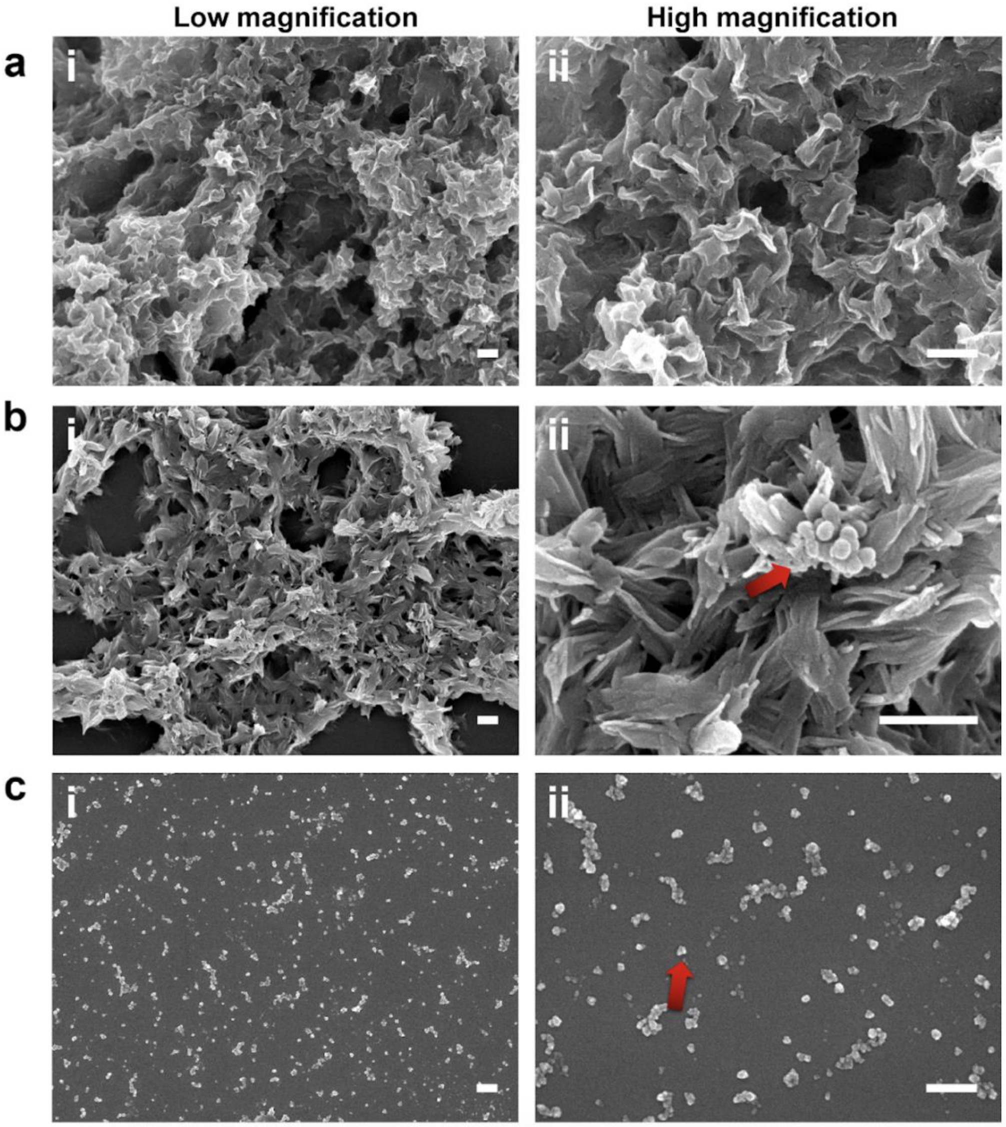

Supplementary Figure 4. SEM of supersaturated CaP mineralization medium stabilized with different concentrations of DNA. a, CaP mineralization medium without addition of DNA. There was ample precipitation of crystalline CaP. **b,** Addition of 50 μg/mL DNA to the mineralization medium. SEM image showed a mixture plate-like crystalline CaP deposits and ACP nanoparticulates (red arrow). The concentration of the DNA was too low to stabilize the supersaturated CaP medium. **c,** Stabilization of the mineralization medium in the form of nanoparticulates (red arrow) after addition of 180 μg/mL DNA to the mineralization medium (bars: 500 nm for all images).

SI-5. SEM images of completely demineralized dentin thin sections that were remineralized using DNA stabilized-CaP mineralization medium for 5 days [Supplementary Figure 5]

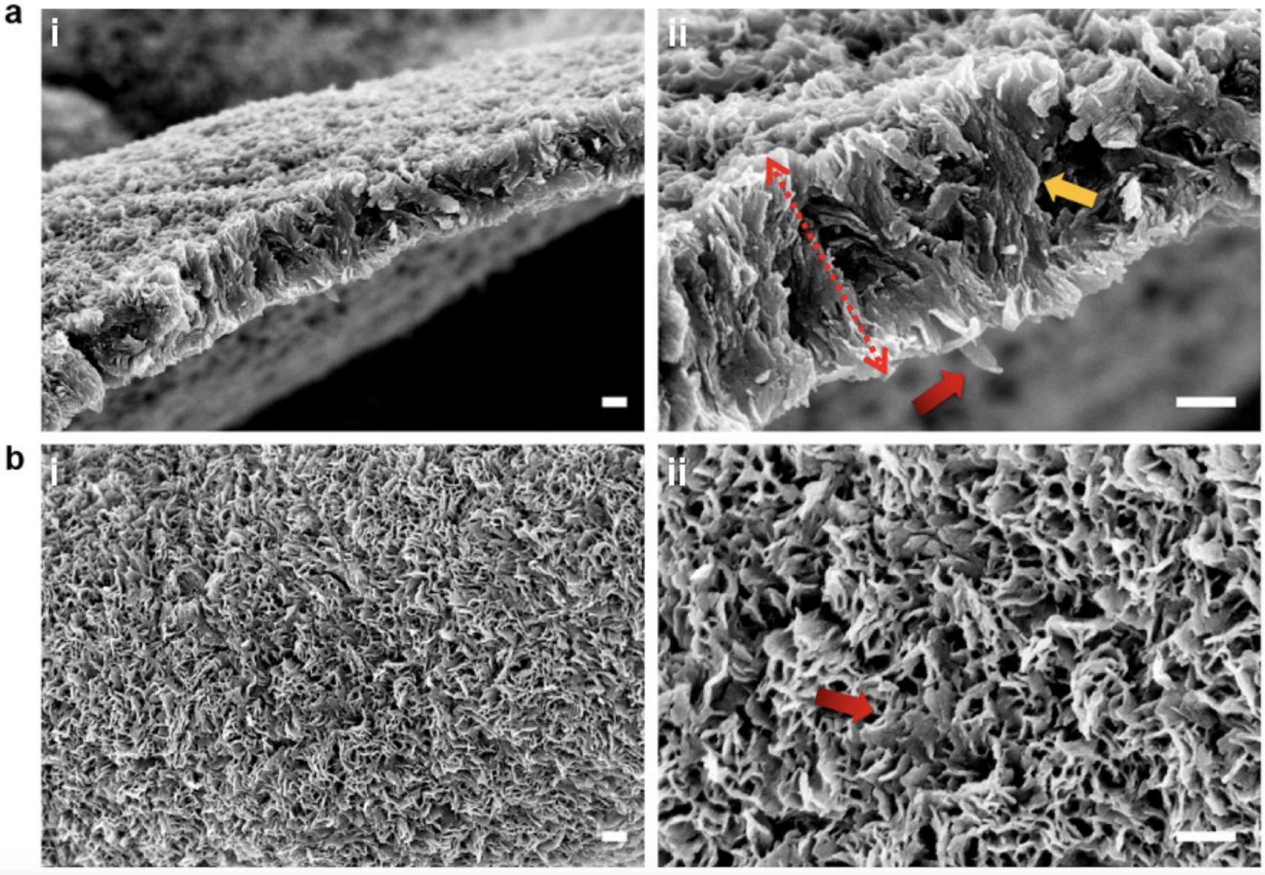

Supplementary Figure 5. SEM images of completely demineralized dentin thin sections that were remineralized using DNA stabilized-CaP mineralization medium for 5 days. a, Sagittal view of remineralized dentin section. **a-ii,** High magnification image of (**a-i**) showing that the thickness of the remineralized dentin was nearly 1 µm (red dashed line), which is much thicker than the original demineralized section (200 nm in thickness). Plate-like crystalline deposits (red arrow) and mineral-infiltrated collagen fibrils (yellow arrow) could be seen. **b,** Coronal view of the remineralized dentin section. **b-ii,** High magnification image of (**b-i**). A lot of mineral platelets could be identified (red arrow) (bar: 250 nm for all images).

SI-6. Characterization of DNA-CaP in the form of amorphous calcium phosphate (ACP) [Supplementary Figure 6]

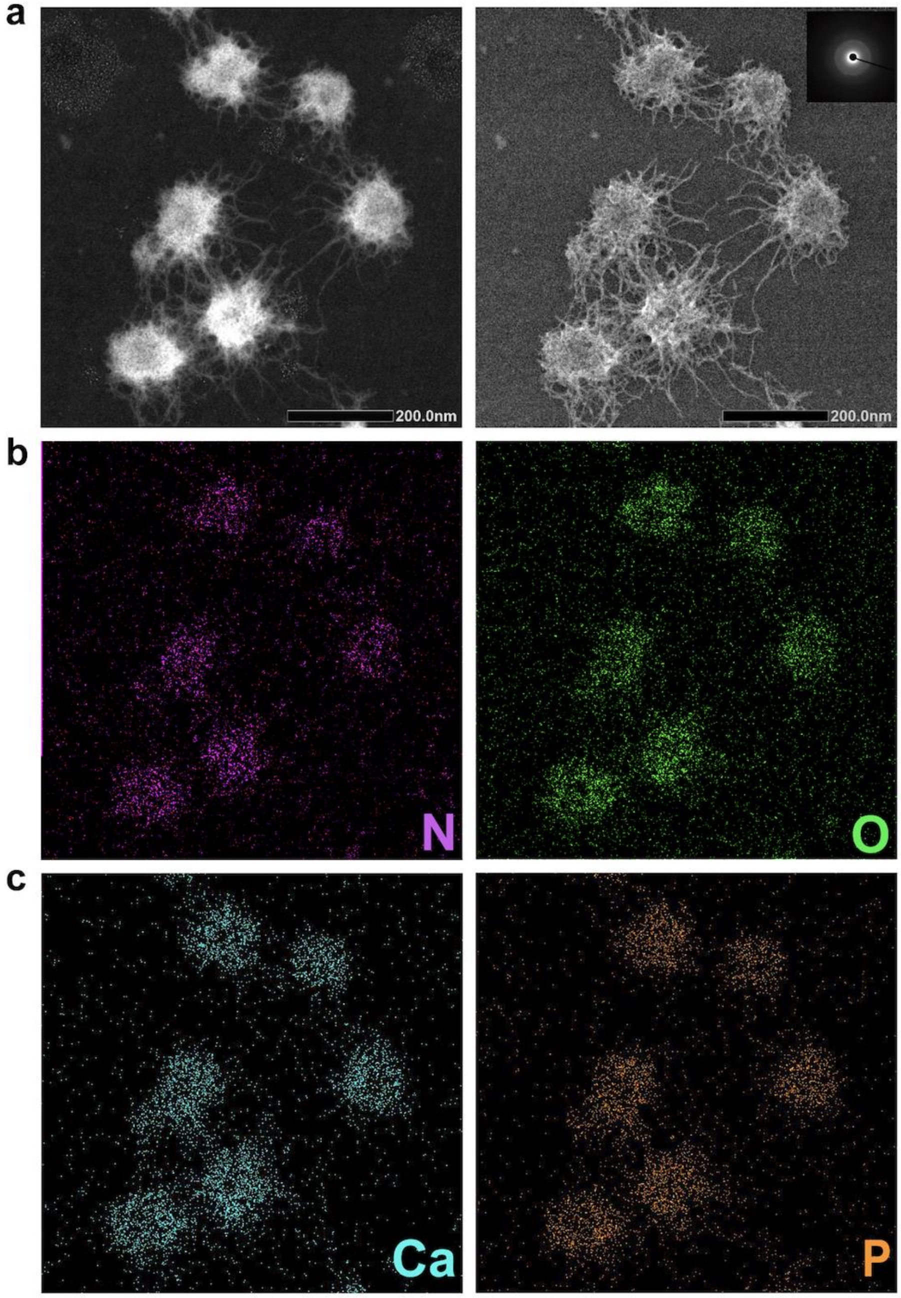

Supplementary Figure 6. Characterization of DNA-CaP in the form of amorphous calcium phosphate (ACP). a, High-angle annular dark-field scanning transmission electron microscopy (HAADF-STEM) images of the roughly spherical nanoparticles that were amorphous in nature, as indicated by lack of discrete rings in the selected are electron diffraction. **b-c,** Elemental mappings of the DNA-ACP nanoparticulates, which were consistent with the presence of nitrogen, oxygen, calcium and phosphorus (bars: 200 nm for all images).

SI-7. Cryogenic electron microscopy (cryo-EM) of DNA-ACP at 8 h after preparation [Supplementary Figure 7]

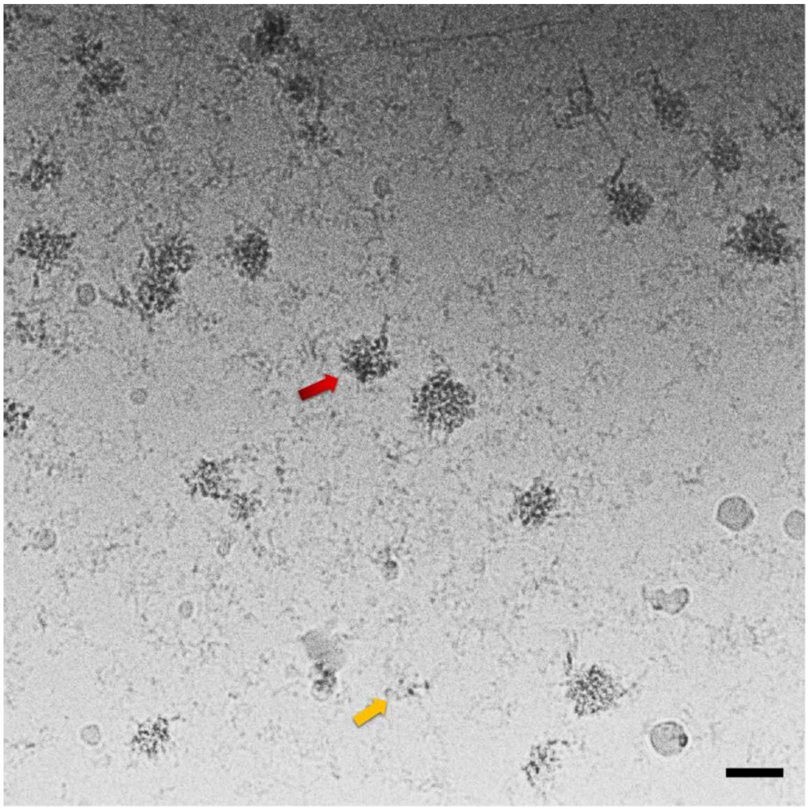

Supplementary Figure 7. Cryogenic electron microscopy (cryo-EM) of DNA-ACP at 8 h after preparation. Electron dense nanoparticles could be identified as DNA-stabilized ACP (red arrow) and DNA-stabilized prenucleation clusters (yellow arrow). Bar: 50 nm.

SI-8. Characterization of single layered collagen fibrils that had been mineralized by DNA-ACP for 5 h [Supplementary Figure 8]

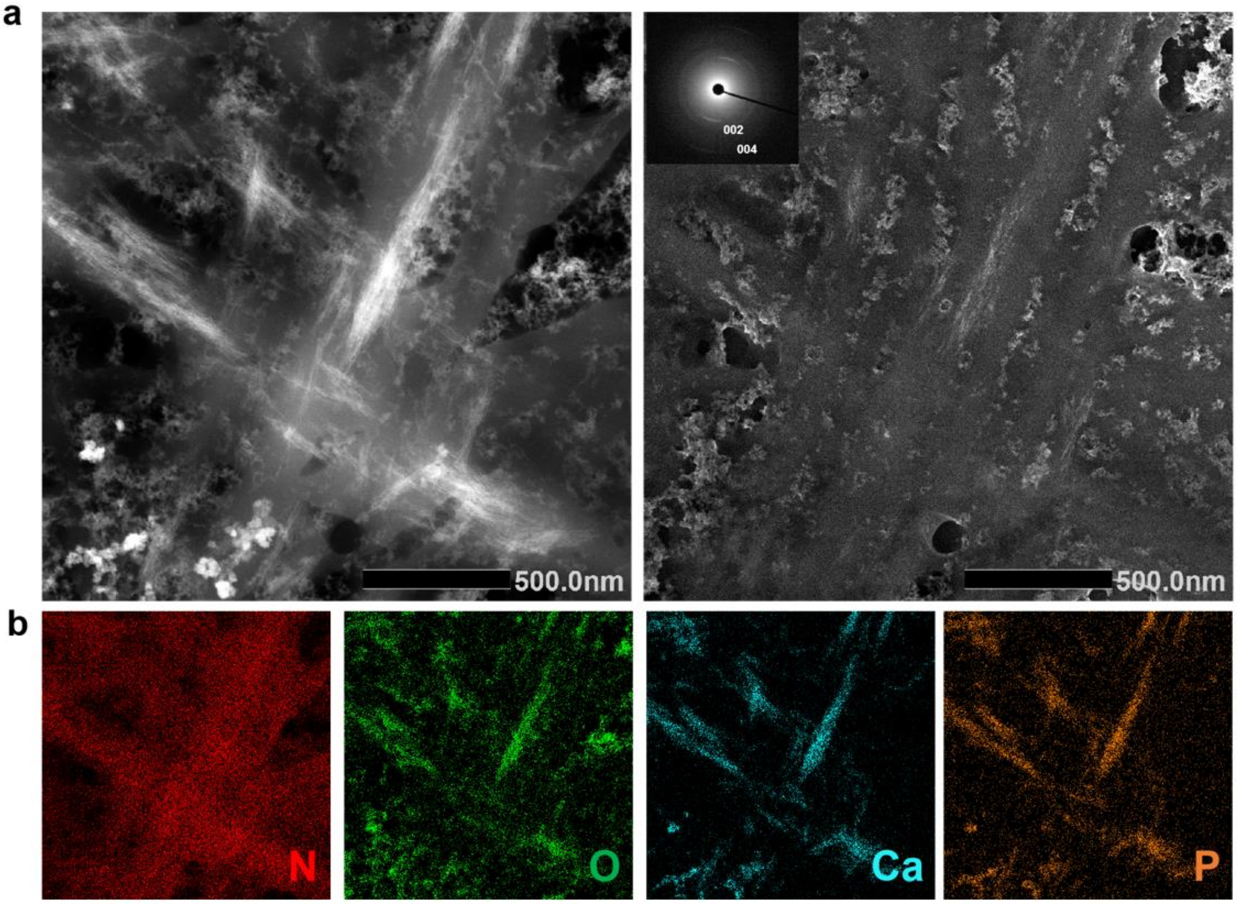

Supplementary Figure 8. Characterization of single layered collagen fibrils that had been mineralized by DNA-ACP for 5 h. a, High-angle annular dark-field scanning transmission electron microscopy (HAADF-STEM) images showed a lot of oriented apatite crystallites (indicated by SAED insert) that were deposited along the c-axis of the collagen fibrils to achieve intrafibrillar mineralization. **b,** Elemental mappings of the mineralized collagen fibrils.

SI-9. Cryo-EM images of collagen fibrils that had been mineralized by DNA-ACP for 24 h [Supplementary Figure 9]

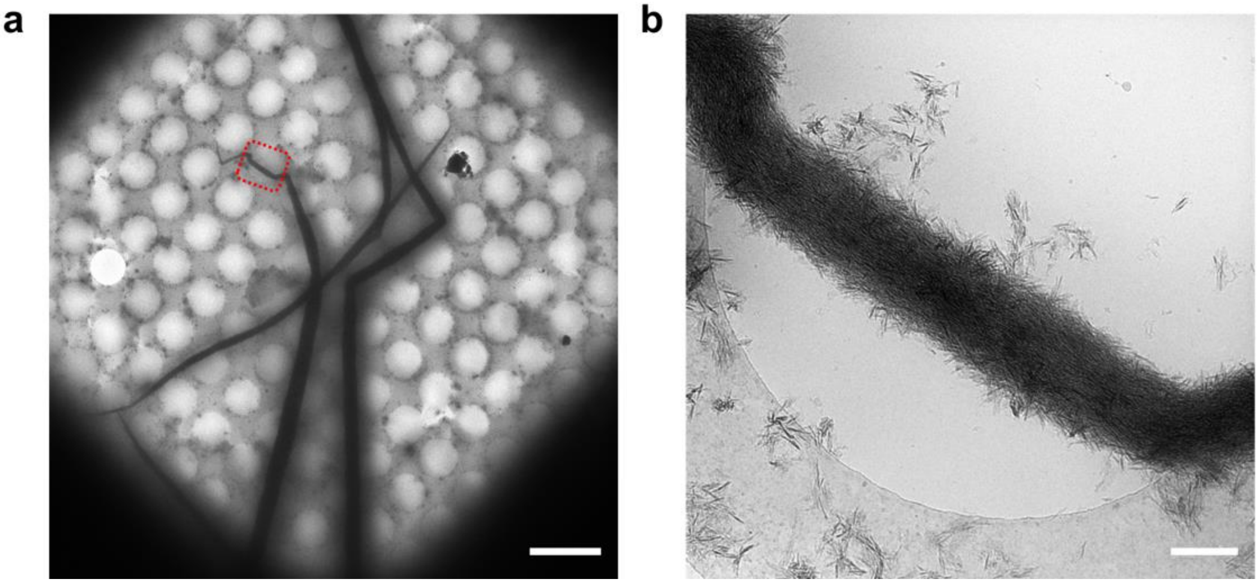

Supplementary Figure 9. Cryo-EM images of collagen fibrils that had been mineralized by DNA-ACP for 24 h. a, Low magnification image of extensively mineralized collagen fibrils (bar: 5 μm). **b,** High magnification of the area indicated by the red rectangle in (**a**). The entire fibril was completely mineralized with intra/extrafibrillar minerals after incubation for 24 hours with DNA-ACP (bar: 250 nm).

SI-10. 3D reconstruction of a collagen fibril completely mineralized by DNA-ACP [Supplementary Figure 10]

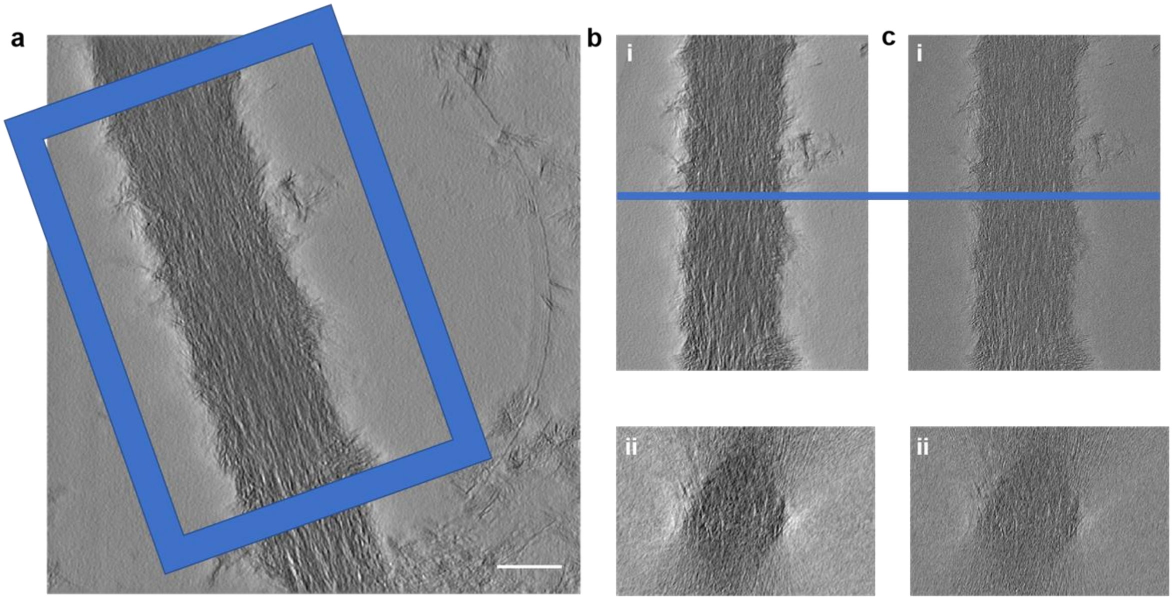

Supplementary Figure 10. 3D reconstruction of a collagen fibril completely mineralized by DNA-ACP. **a,** The reconstructed fibril was rotated by self-written program in Matlab R2020a (bar: 250 nm). **b-c,** Images were taken from 600*800*392 out of 1024*1024*392 (bin 4). Images in (**b**) were Gaussian filtered to 10A. **b-ii and c-ii** are images sectioned virtually at the blue line (i.e. half-y plane of the 600*800*392 reconstruction).

SI-11. Binding of DNA with collagen fibrils [Supplementary Figure 11]

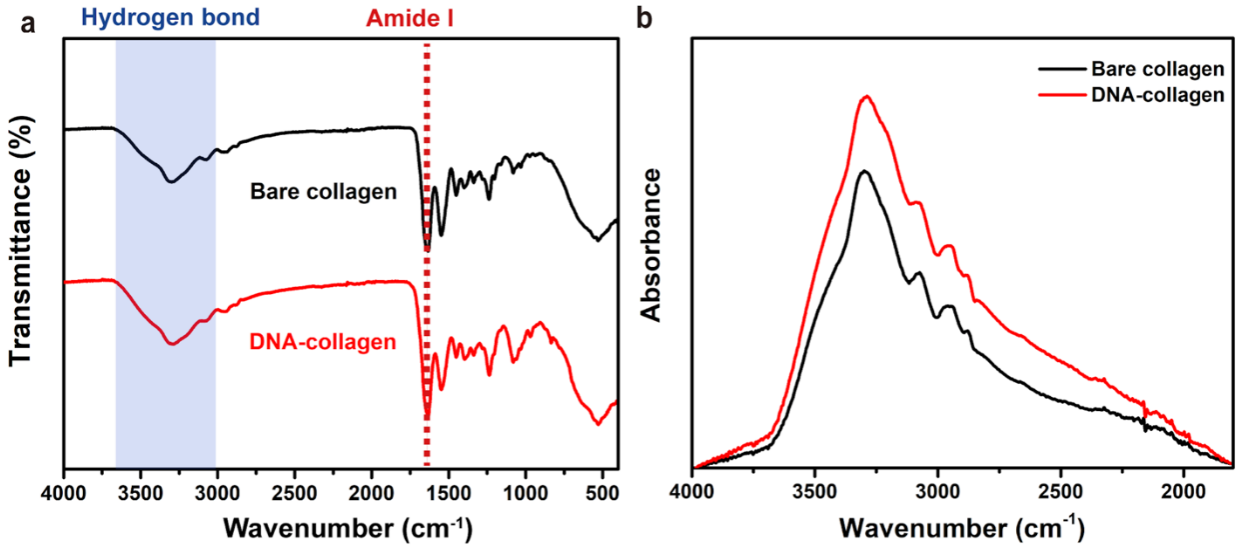

Supplementary Figure 11. Binding of DNA with collagen fibrils. a, Infrared spectra of pristine collagen (black) and DNA-bound collagen (red). Peaks at 3,000-3,700 cm^{-1} were assigned to hydrogen bonds. The stretching vibration of amide I (1,650 cm^{-1}) of the collagen fibrils was still present after binding of nucleic acids. This indicates that DNA-collagen still possess a triple helix structure. **b,** Infrared spectra of pristine collagen (black) and DNA-bound collagen (red). Peaks at 3,000 cm^{-1}-3,700 cm^{-1} were assigned to the hydrogen bond. The absorption band in this region broadened in DNA binding collagen samples, indicating the formation of hydrogen bond between nucleic acids and collagen fibrils.

SI-12. Conventional TEM of collagen mineralization induced by DNA with different molecular weight [Supplementary Figure 12]

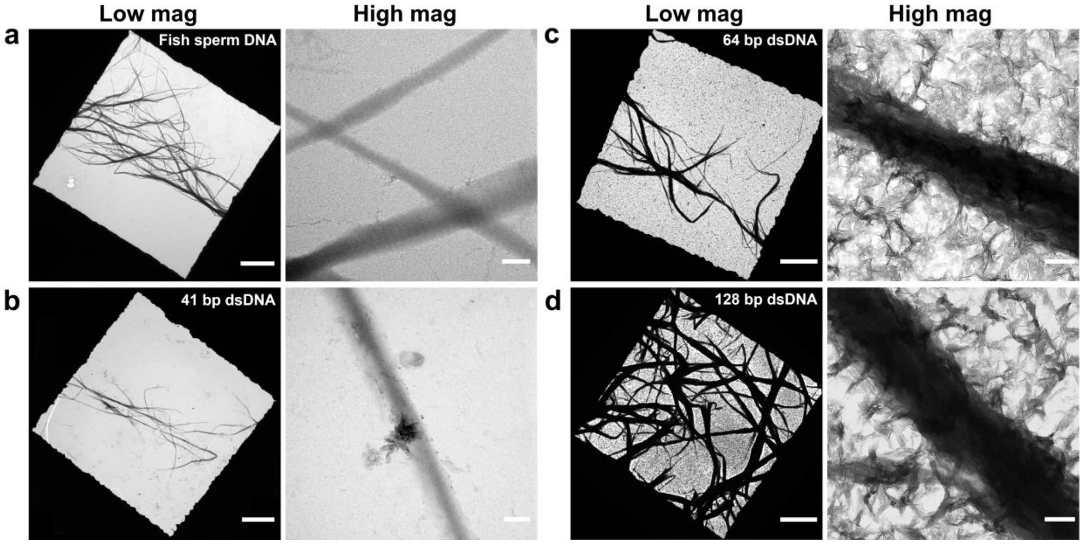

Supplementary Figure 12. TEM of collagen mineralization induced by DNA with different molecular weight. a, Grids coated with collagen fibrils were immersed in fish sperm DNA-calcium phosphate (CaP) containing medium for 5 days. Collagen fibrils did not achieve intrafibrillar mineralization. **b,** Collagen fibrils immersed in 41 bp dsDNA-CaP containing medium for 5 days. Minerals deposited on the surface of the fibrils. **c,** Collagen fibrils immersed in 64 bp dsDNA-CaP containing medium for 5 days. Intrafibrillar/extrafibrillar mineralization were achieved in this medium. **d,** Collagen fibrils immersed in 128 bp dsDNA-CaP containing medium for 5 days. Collagen fibrils were all extensively mineralized. Left: low magnification of collagen fibrils (bar: 10 μm). Right: high magnification of collagen fibrils (bar: 250 nm).

SI-13. Molecular dynamics (MD) simulation of the mechanism for DNA-ACP formation [Supplementary Figure 13]

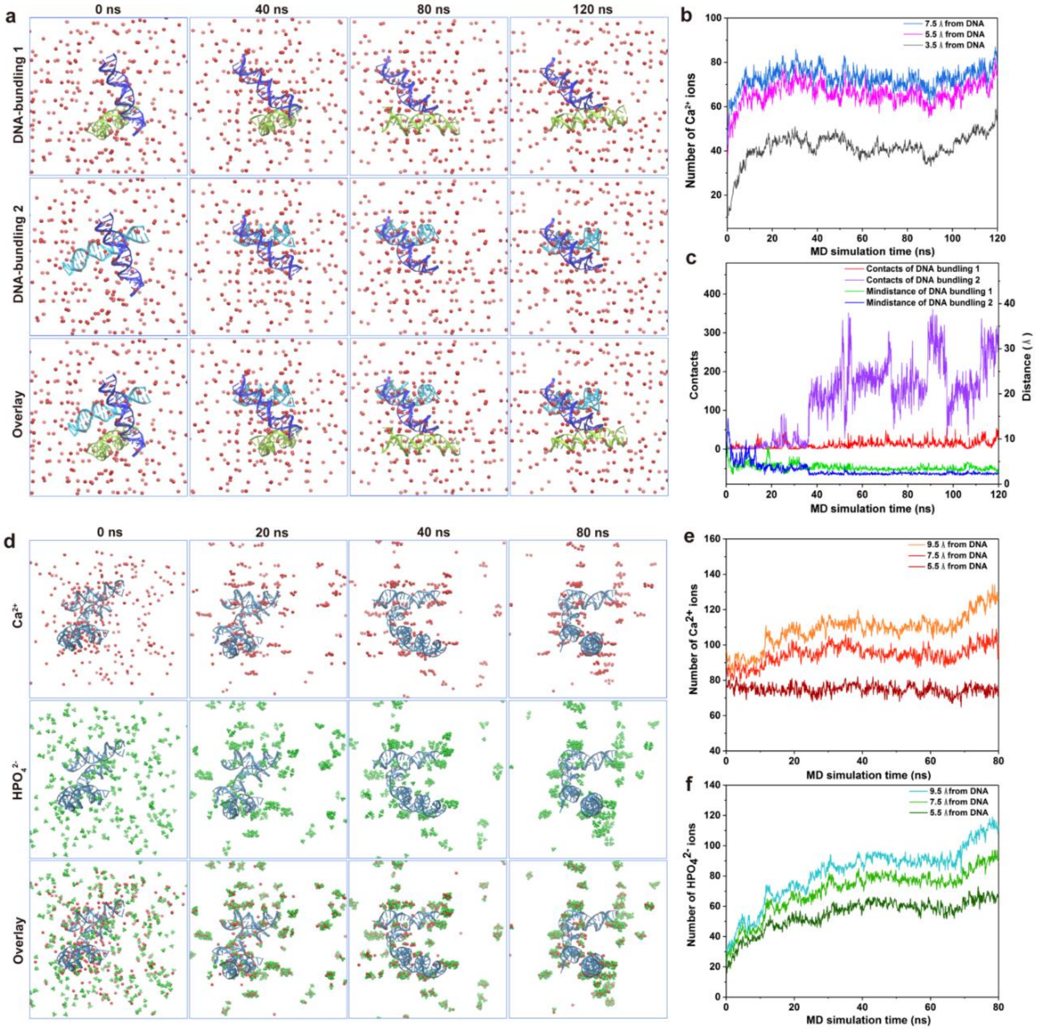

Supplementary Figure 13. Molecular dynamics (MD) simulation of the mechanism for DNA-ACP formation. a, All-atom MD simulations of three DNA double chains (21 base pairs) placed in a box (a = 12.8 nm, b = 13.2 nm, c = 13.7 nm, α = 90°, β = 90°, γ = 90°) and the centroid distance of DNA was 10 Å. Snapshots of MD trajectory showed green/blue DNA chains approximated the purple DNA chains to form DNA bundling in the presence of Ca^{2+} (red dots). **b,** Variations in the distribution of Ca^{2+} away from DNA at 7.5 Å, 5.5 Å and 3.5 Å. The number of Ca^{2+} increased with simulation time, indicating DNA attracting Ca^{2+}. **c,** Contact numbers of atoms in DNA bundling increased and mindistance of DNA bundling decreased, indicating DNA aggregation and condensation. **d,** DNA aggregation in the presence of Ca^{2+} and HPO_4^{2-} (a = 15.7 nm, b = 16.1 nm, c = 16.5 nm). Snapshots of MD trajectory showing gathering of Ca^{2+} and HPO_4^{2-} around the DNA to form DNA-ACP. **e-f,** Variations in the distribution of Ca^{2+} (**e**) and HPO_4^{2-} (**f**) at 9.5 Å, 7.5 Å and 5.5 Å from DNA. The number of both ions increased with simulation time.

SI-14. Captions of Supplementary Movies 1-5

Supplementary Movie 1. 3D reconstruction from a confocal laser scanning microscopy showing extracellular DNA deposited in mineralized nodules secreted by mouse vascular smooth muscle cells cultured in osteogenic medium *in vitro.*

Supplementary Movie 2. Cryogenic electron microscopy showing intrafibrillar and extrafibrillar mineralization of the entire collagen fibril was accomplished at 24 h.

Supplementary Movie 3. 3D reconstruction from a cryogenic electron tomography tilt series showing a type I collagen fibril achieved complete mineralization induced by DNA-ACP at 24 h.

Supplementary Movie 4. MD simulations of DNA aggregation and condensation by introducing calcium ions to nucleic acids.

Supplementary Movie 5. MD simulations of DNA-ACP formation process.

SI-15. Table S1. DNA sequence [Supplementary Table 1]

Structure	No.	Sequence (5'-3')
dsDNA	41 bp	GTAAAACGACGGCCAGTGTTTTCATGGTCATAGCTGTTTCC
	64 bp	GTAAAACGACGGCCAGTGTTTTTTTTTTTTTTTTTTTTTTTTTTTCATGGTC<br>ATAGCTGTTTCC
	128 bp	GTAAAACGACGGCCAGTGTTTTTTTTTTTTTTTTTTTTTTTTTTTTTTTTTTT<br>TTTTTTTTTTTTTTTTTTTTTTTTTTTTTTTTTTTTTTTTTTTTTTTTTTTTT<br>CATGGTCATAGCTGTTTCC

SI-16. Supplementary references

[44]T. Iwayama, T. Okada, T. Ueda, K. Tomita, S. Matsumoto, M. Takedachi, S. Wakisaka, T. Noda, T. Ogura, T. Okano, P. Fratzl, T. Ogura, S. Murakami, *Sci. Adv.* **2019**, *5,* eaax0672.

[45]J. Wang, N. Fang, J. Xiong, Y. Du, Y. Cao, W.-K. Ji, *Nat. Commun.* **2021**, *12*, 1252.

[46]C. A. Gregory, W. G. Gunn, A. Peister, D. J. Prockop, *Anal. Biochem.* **2004**, *329*, 77.

[47]N. Kakudo, K. Kusumoto, A. Kuro, Y. Ogawa, *Wound Repair Regen.* **2006**, *14*, 336.

[48]H. Yuan, C. A. van Blitterswijk, K. de Groot, J. D. de Bruijn, *Tissue Eng.* **2006**, *12*, 1607.

[49]S. Orzechowska, R. Świsłocka, W. Lewandowski, *Materials (Basel)*. **2020**, *13*, 2130.

[50] M. Shen, M. Lin, M. Zhu, W. Zhang, D. Lu, H. Liu, J. Deng, K. Que, X. Zhang, *Biochim. Biophys. Acta - Gen. Subj.* **2019**, *1863*, 167.

[51] S. Q. Zheng, B. Keszthelyi, E. Branlund, J. M. Lyle, M. B. Braunfeld, J. W. Sedat, D. A. Agard, *J. Struct. Biol.* **2007**, *157*, 138.

[52] J. R. Kremer, D. N. Mastronarde, J. R. McIntosh, *J. Struct. Biol.* **1996**, *116*, 71.

[53] Q. Song, K. Jiao, L. Tonggu, L. G. Wang, S. L. Zhang, Y. D. Yang, L. Zhang, J. H. Bian, D. X. Hao, C. Y. Wang, Y. X. Ma, D. D. Arola, L. Breschi, J. H. Chen, F. R. Tay, L. N. Niu, *Sci. Adv.* **2019**, *5*, eaav9075.

[54] L. Wang, F. Yan, *J. Chem. Inf. Model.* **2018**, *58*, 2123.

[55] H. Sun, Y. Li, S. Tian, L. Xu, T. Hou, *Phys. Chem. Chem. Phys.* **2014**, *16*, 16719.
